# Interactive effects of genotype with prenatal stress on DNA methylation at birth

**DOI:** 10.1038/s41380-025-03312-6

**Published:** 2025-10-24

**Authors:** Rosa H. Mulder, Vilte Baltramonaityte, Serena Defina, Katerina Trajanoska, Matthew Suderman, Emanuel Schwarz, Marco P. M. Boks, Esther Walton, Charlotte A. M. Cecil, Janine F. Felix

**Affiliations:** 1 Department of Adolescent and Child Psychiatry and Psychology, Erasmus MC, University Medical Center Rotterdam, Rotterdam, The Netherlands; 2 Generation R Study Group, Erasmus MC, University Medical Center Rotterdam, Rotterdam, The Netherlands; 3 Department of Psychology, University of Bath, Bath, UK; 4 Department of Internal Medicine, Erasmus MC, University Medical Center Rotterdam, Rotterdam, The Netherlands; 5 MRC Integrative Epidemiology Unit, Population Health Sciences, Bristol Medical School, University of Bristol, Bristol, UK; 6 Hector Institute for Artificial Intelligence in Psychiatry, Central Institute of Mental Health, Medical Faculty Mannheim, Heidelberg University, Mannheim, Germany; 7 Department of Psychiatry and Psychotherapy, Central Institute of Mental Health, Medical Faculty Mannheim, Heidelberg University, Mannheim, Germany; 8 German Center for Mental Health (DZPG), Mannheim, Germany; 9 Department of Psychiatry, Amsterdam UMC, Amsterdam, The Netherlands; 10 Dimence Institute for Specialized Mental Health Care, Dimence Group, Deventer, The Netherlands; 11 Department of Epidemiology, Erasmus MC, University Medical Center Rotterdam, Rotterdam, The Netherlands; 12 Molecular Epidemiology, Department of Biomedical Data Sciences, Leiden University Medical Center, Leiden, the Netherlands; 13 Department of Pediatrics, Erasmus MC, University Medical Center Rotterdam, Rotterdam, The Netherlands

**Keywords:** Genetics, Predictive markers

## Abstract

Intrauterine stress exposure is associated with offspring health. DNA methylation (DNAm) is a putative underlying mechanism, but large population-based studies reported limited associations between prenatal stress and DNAm. Recent research has shown that environmental factors *in interaction* with genetic variants are better predictors of DNAm than environment or genotype alone. We investigated whether interactions of maternal prenatal stress with genetic variants are associated with DNAm at birth. We examined 2963 mother-child pairs from the population-based Generation R Study and Avon Longitudinal Study of Parents and Children, using a harmonized, comprehensive cumulative prenatal stress measure. We tested genome-wide genotype-by-prenatal stress interactions on epigenome-wide DNAm (GxEmodel), and models including only genetic variants (Gmodel) or prenatal stress (Emodel) as predictors. Follow-up analyses included Gene Ontology analyses and mediation analyses of prenatal alcohol intake, smoking, gestational age, and birth weight. We report two independent gene-by-prenatal-stress interactions on DNAm after multiple testing correction, including five genetic variants in *CHD2* and *ORC5*, and two DNAm sites in *EPPK1*. By comparison, the Gmodel showed 691,202 associations and the Emodel showed three associations in genes *AHRR, GFI1*, and *MYO1G*, which could largely be explained by prenatal smoking. Genes linked to suggestive GxEmodel results were often involved in neuronal development. Our results provide some support of interaction of prenatal stress with the child’s genome on DNAm of genes related to neuronal development. Based on these models, genetic main effects on DNA methylation at birth were much more abundant than gene-by-prenatal stress interactions were.

## Introduction

*In utero* stress exposure has been associated with adverse offspring mental and physical health outcomes, including internalizing symptoms [1], adiposity [2], asthma, and allergies [3], and has been hypothesized to put children in a disadvantaged position from early life onwards. Differential DNA methylation (DNAm) has been suggested as a putative mechanism underlying these associations, as DNAm has been linked to prenatal exposures such as maternal smoking [4], postnatal psycho-social stress [5], and to child outcomes such as body mass index [6], asthma [7] and cortisol reactivity [8]. Several multi-cohort studies have probed epigenome-wide associations of maternal prenatal stress with offspring DNAm, with varying results [9–11]. The largest study to date, including 5496 children from 12 cohorts, reported limited associations for DNAm sites located in genes that have been implicated in neurodegeneration, immune and cellular functions, and epigenetic regulation [10].

A growing body of research, however, shows that environmental factors *in interaction with* genetic variants are better predictors of DNAm than environmental factors or genetic variants alone, when looking into CpG sites within variably methylated regions (VMRs). For example, Teh, Pan [12] studied genome-wide interactions of 19 prenatal factors, including gestational age, maternal smoking and maternal depression, on highly variable neonatal DNAm sites. For 75% of the sites, DNAm was better predicted by the interaction between genotype and the environment than by either genotype or environment alone. Environment-only was never the best predictor of DNAm in that study. Similarly, a study by Czamara, Eraslan [13] in four cohorts, examining 10 prenatal factors, showed that gene-environment interactions best predicted DNAm in variably methylated regions in 38-60% of analyses, while genotype-only models were best in 11-30% and environment-only models were best in only <1-4%. However, while these studies analyzed which *type* of model worked best, they did not aim to identify specific genetic variants, environmental variables or DNAm sites. Knowing which genetic variants interact with prenatal stress in relation to DNA methylation would help to better understand the biological pathways underlying the gene-environment effects on health.

We therefore aimed to study genome-wide interactions between genetic variants and cumulative prenatal stress in relation to epigenome-wide DNAm at birth. We also aimed to test the hypothesis that DNAm is better predicted by the interaction of genetic variants and stress than by either factor alone. We used a comprehensive cumulative measure of psycho-social maternal stress during pregnancy, which has previously been related to suboptimal neurodevelopmental, mental, and cardiovascular outcomes [14–16]. We meta-analyzed data from two population-based cohorts, the Generation R Study in the Netherlands (Generation R) and the Avon Longitudinal Study of Children and Parents (ALSPAC) in the United Kingdom and followed up associations to study unique stress domain contributions, as well as running mediation analyses of maternal prenatal smoking, alcohol use, gestational age and birth weight. Lastly, we performed enrichment analyses to gain insight into potential biological pathways.

## Methods

### Setting

We used three non-overlapping datasets from two prospective population-based cohorts: two datasets from Generation R and a third dataset from ALSPAC.

In the Generation R Study, pregnant women residing in the study area of Rotterdam in the Netherlands with an expected delivery date between April 2002 and January 2006 were invited to participate in the study [17]. The Generation R Study is conducted in accordance with the World Medical Association Declaration of Helsinki and has been approved by the Medical Ethics Committee of Erasmus MC, Rotterdam. Informed consent was obtained for all participants.

In ALSPAC, pregnant women resident in Avon, UK with expected dates of delivery between 1^st^ April 1991 and 31^st^ December 1992 were invited to take part in the study [18, 19]. The ALSPAC website contains details of all the data that are available through a fully searchable data dictionary and variable search tool (http://www.bristol.ac.uk/alspac/researchers/our-data/). Ethical approval for the ALSPAC study was obtained from the ALSPAC Ethics and Law Committee and the Local Research Ethics Committees. Informed consent for the use of data collected via questionnaires and clinics was obtained from participants following the recommendations of the ALSPAC Ethics and Law Committee at the time. Consent for biological samples has been collected in accordance with the Human Tissue Act (2004).

### Study Population

The full selection procedure is described in the Supplemental Information. In Generation R, 9778 pregnant mothers gave birth to 9749 live-born children and in ALSPAC, the initial number of pregnancies enrolled was 14,541. Participants were selected based on availability of genetic data (*n*_Generation R_ = 7502; *n*_ALSPAC_ = 8797), as well as cumulative prenatal stress information (*n*_Generation R_ = 5684; *n*_ALSPAC_ = 7483), and DNAm data as measured with the Infinium HumanMethylation450 BeadChip (Illumina Inc., San Diego, CA) in Generation R (*GENR 450 K*) and ALSPAC (*ALSPAC 450 K*) or with the Infinium MethylationEPIC v1.0 Beadchip in Generation R (*GENR EPIC*) (*n*_GENR 450K_ = 1231; *n*_GENR EPIC_ = 986; *n*_ALSPAC_ = 793). Additionally, one of each pair of children with cryptic relatedness (IBD > 0.15) were removed, based on data availability or otherwise randomly. As a result, *GENR 450 K* included 1224 children, *GENR EPIC* included 949, and *ALSPAC 450 K* included 790 – for a total of 2963 children.

### Genotyping

In the Generation R Study, children were genotyped with the Illumina HumanHap 610 or 660 quad chips. A full description has been published previously [20]. Data were imputed to the 1000 genomes reference panel (Phase 1 version 3). Phasing was done using MACH software, and imputation using Minimac software. The ALSPAC children have been genotyped with the Illumina HumanHap 550 quad chip [21]. The data were imputed to a phased version of the 1000 genomes references panel (Phase 1 version 3) from the Impute2 reference data repository.

In all (sub-)cohorts, we used best-guess genotypes. Quality control was done with PLINK 1.90 [22]. Autosomal variants were selected and variants with SNP call rates of <95%, with evidence for violation of Hardy-Weinberg equilibrium (*p* < 1×10^-07^), with a minor allele frequency <5%, or with low imputation quality (Rsq<0.3 in Generation R and info scores <0.8 in ALSPAC, according to local practices [20, 21]) were removed. Insertions, deletions, and multi-allelic positions were also removed. Samples were excluded in the case of sex mismatches, minimal or excessive heterozygosity, or a sample call rate of <97.5%.

This quality control procedure resulted in 5,584,862 SNPs in *GENR 450 K*; 5,627,497 SNPs in *GENR EPIC;* and 5,797,754 SNPs in *ALSPAC 450 K*. To reduce the multiple testing burden, SNPs were pruned based on linkage disequilibrium and haplotype blocks (window size=50 SNPs, step size=5 SNPs, VIF = 2) in the largest subcohort, *GENR 450 K*, which resulted in 447,713 SNPs. Of these, a final set of 374,152 SNPs was common to all three (sub-)cohorts.

### Cumulative prenatal stress

The cumulative prenatal stress score was computed from ~50 stress-related items measured during pregnancy. The full item list and a detailed description of the score calculation can be found elsewhere (https://github.com/SereDef/cumulative-ELS-score [15, 16]). Briefly, in order to maximize data harmonization across cohorts, stress items were selected (based on closest item-similarity), dichotomized (0 = no risk; 1 = risk) and assigned to one of four stress domains: life events (e.g. death of a relative), contextual risk (e.g. financial problems), personal stress (e.g. depression), and interpersonal stress (e.g. family conflict). Stress domain scores (ranging from 0 to 1) were then computed by averaging items within each domain. A total prenatal stress score was obtained by summing all domain scores (range: 0 to 4). Individuals with >50% of all stress items missing were excluded. Missing data were imputed at the individual item level using predictive mean matching with 60 iterations, as implemented by the *mice* package [23] in R version 4.0 [24]. Within the selected samples of each (sub-)cohort, cumulative prenatal stress scores were standardized. To reduce the influence of extreme outliers, we winsorized values outside the range of (25^th^ percentile - 3*interquartile range (IQR)) to (75^th^ percentile + 3*IQR).

### DNA methylation

For both cohorts, DNA extracted from cord blood was bisulfite converted. Samples were processed with the Illumina Infinium HumanMethylation450 BeadChip (Illumina Inc., San Diego, CA) in *GENR 450 K* and *ALSPAC 450 K* and with the Infinium MethylationEPIC v1.0 Beadchip in *GENR EPIC*.

In *GENR 450 K* and *GENR EPIC*, the CPACOR workflow [25] was applied for quality control. Arrays with observed technical problems such as failed bisulfite conversion, hybridization or extension as well as arrays with a sex mismatch were removed. Arrays with a call rate >95% per sample were carried forward into normalization.

In *ALSPAC 450 K*, quality control was done using the *meffil* package [26] in R version 3.4.3. Samples with mismatched genotypes, mismatched sex, incorrect relatedness, low concordance with samples collected at other time points, extreme dye bias and poor probe detection were removed and carried before normalization.

In order to minimize cohort effects, the data from *GENR 450 K and ALSPAC 450 K* have been previously normalized as a single dataset [27] and data from *GENR EPIC* were normalized using the same procedure. Functional normalization was performed (using 10 control probe principle components with slide included as a random effect) with the *meffil* package in R [26]. Probes were excluded if they had a detection *p* > 0.01 or low bead count (<3) in >10% of the samples. In total, 472,450 autosomal methylation sites (CpGs) passed these quality control filters in *GENR 450 K* and *ALSPAC 450 K*. To reduce the computational burden of the genome-wide analyses on the methylome, only probes that were previously identified as having epigenome-wide significant (*p* < 1×10^-7^) inter-individual variation in DNAm at birth in these cohorts were carried forward into analyses, leaving 100,687 CpGs [27]. Of these, cross-reactive probes (*n* = 14,451 CpGs) were removed [28]. Last, only probes present and passing quality control on both array types were selected, resulting in a final set of 86,236 CpGs (sample-size weighted median MAD-[median absolute deviation]-score [IQR] = 0.04 [0.03-0.06]; range=0.001-0.641). DNAm levels were represented as beta values, indicating the ratio of methylated signal relative to the sum of methylated and unmethylated signal per CpG. To reduce the influence of extreme outlying values, beta values of each CpG outside the range of (25^th^ percentile - 3*interquartile range (IQR)) to (75^th^ percentile + 3*IQR) were winsorized.

### Covariates

All models were adjusted for sex of the child as determined at birth, the first 5 genetic principle components, estimated blood cell composition (CD4 + T-lymphocytes, CD8 + T-lymphocytes, natural killer cells, B-lymphocytes, monocytes, granulocytes, and nucleated red blood cells) as based on a cord blood reference panel [29], and DNAm batch effects (25 sample plates in *GENR 450 K*, 12 sample plates in *GENR EPIC*, and 20 surrogate variables in *ALSPAC 450 K* [26, 30]).

### Statistical analyses

Analyses were performed with an adapted version [16] of the *GEM* software package [31] in R [24] version 4.0.5. This package applies large matrix operations allowing for fast analysis of genome-wide SNPs and CpGs and enabling us to test the following models: (i) a *GxEmodel* – our primary model of interest – in which the interaction effect of each SNP with cumulative prenatal stress was iteratively regressed on DNAm at each CpG site. For comparative purposes, we also tested (ii) a *Gmodel*, in which each SNP was iteratively regressed on DNAm at each CpG site, and (iii) an *Emodel*, in which cumulative prenatal stress was iteratively regressed on DNAm at each CpG site. A dominant model was applied to the GxEmodel and Gmodel, meaning that heterozygous and homozygous minor genotypes were contrasted against homozygous major genotypes, in order to make the models more robust against outlying values. The three models were performed in each (sub-)cohort separately, and results were meta-analyzed using inverse-variance weighted fixed effects with METAL [32]. The significance threshold of the meta-analyses was Bonferroni-corrected for the number of tests. For the GxEmodel and Gmodel (*n*_*SNPs*_ = 374,152, *n*_*CpGs*_ = 86,236: 32,265,371,872 tests) the threshold was set to *p* < 1.55×10^-12^, and for the Emodel (86,236 tests), the threshold was set to *p* < 5.80×10^-07^. To assess heterogeneity between (sub-)cohort results, the I^2^ statistic was used, with 75% taken as indication of considerable heterogeneity [33].

### Model comparisons

First, we compared the occurrence of *cis-* versus *trans*-effects among suggestive findings (p < 5×10-08; i.e. genome-wide threshold) in the Gmodel and the GxEmodel. Second, earlier studies tested SNP-by-environment interactions with CpGs only among SNPs or CpGs for which an association was found in a genotype-only model. We tested whether SNPs/CpGs that showed suggestive associations in the Gmodel or Emodel had a higher chance of being part of a suggestive association in the GxEmodel. Associations in the Gmodel and the GxEmodel with *p* < 5×10^-08^ (i.e. genome-wide threshold), and associations in the Emodel with *p* < 1×10^-05^ were considered suggestive. We performed enrichment analyses comparing suggestive versus non-suggestive SNPs and CpGs using Fisher’s exact tests (significance threshold: *p* < 0.05).

As a sensitivity analysis, we performed similar enrichment analyses to assess if suggestive SNPs and CpGs had been identified in an earlier large-scale methylation quantitative trait locus (meQTL) study [34].

### Follow-up analyses

Multiple follow-up analyses were performed. Since the Gmodel has been tested extensively previously in search of methylation quantitative trait loci (meQTLs) [21, 34], we only followed-up results from the GxEmodel and Emodel. First, we looked up significant CpGs in the GxEmodel and/or Emodel in the EWAS Catalog for previously reported associations [35]. Second, we looked up genes annotated to significant SNPs and CpGs in the GxEmodel and/or Emodel via phenome-wide association studies (PheWASs) using the online GWAS Atlas tool (https://atlas.ctglab.nl/PheWAS) [36], including 4756 GWASs, using a Bonferroni corrected *p* < 1.05×10^-05^. Annotation of SNPs and CpGs was performed using ANNOVAR linking variants reported in the 1000 Genomes Project and SNPdb [37] and the Illumina HumanMethylation450 v1.2 Manifest (Illumina Inc.), respectively. Third, significant associations in the GxEmodel (*p* < 1.55×10^-12^) and/or Emodel (*p* < 5.80×10^-07^) were followed up with linear regressions containing the effects of the four stress domains (life events, contextual risk, personal stress, and interpersonal stress) in one model to identify unique associations of each of these stress types, independent of the other types. Here, associations with *p* < 0.05 were interpreted as a unique contribution to the GxE association on DNAm for that stressor. Fourth, significant associations in the GxEmodel and/or Emodel were tested for potential mediation of prenatal stress effects on DNAm by maternal prenatal smoking, maternal prenatal alcohol intake, gestational age, and birth weight (each modeled separately), using the Lavaan package in R [38]. Mediation was deemed to be significant if the AB path (predictor -> mediator, mediator -> outcome) has a *p*-value below a Bonferroni-corrected threshold of 0.0125 (corrected for the number of mediators). Last, functional enrichment analysis of associated biological pathways was performed with Gene Ontology for models for which results could not be explained by a mediator. Genes annotated to suggestive unique SNPs and CpGs were interrogated using the GOfuncR package [39] in R. The GOfuncR package compares associated pathways of candidate versus background genes using hypergeometric testing and a family-wise error rate correction for multiple testing. Genes annotated to all non-suggestive SNPs and CpGs included in the main analyses were used as the background set.

### Mediators

In Generation R, mothers reported on prenatal tobacco smoking and alcohol consumption via questionnaires in the first, second, and third trimester. In ALSPAC, mothers reported via questionnaires on tobacco smoking in the second and third trimester and on alcohol consumption in the first and second trimester. For both cohorts, gestational age at birth was determined using fetal ultrasound examinations or last menstrual period, and birth weight was obtained from midwife and hospital registries.

## Results

*GENR 450 K*, *GENR EPIC*, and *ALSPAC 450K* included 49.9%, 47.7%, and 49.1% boys, and mothers were 32.2, 32.0, and 29.7 years old at birth, respectively. After winsorizing, mean cumulative prenatal stress scores were 0.36 (SD = 0.28, min=0.00, max=1.48), 0.44 (SD = 0.35, min=0.00, max=1.48), and 0.51 (SD = 0.28, min=0.00, max=1.69), respectively, with a theoretical maximum score of 4 (Supplemental Fig. 1). There were differences between the (sub-)cohorts, as cumulative prenatal stress was higher on average in ALSPAC than in the GENR subcohorts (specifically contextual risk and personal risk; life events and interpersonal risk were highest in GENR EPIC), gestational age and weight at birth were somewhat lower in ALSPAC, as was maternal age at birth (Table 1).

**Table 1:** Sample characteristics.

	*GENR 450 K*	*GENR EPIC*		*ALSPAC 450 K*	p*
*N*	1224	949		790	
Cumulative prenatal stress (mean (SD))	0.36 (0.28)	0.44 (0.35)		0.51 (0.28)	<0.001
Life events domain (mean (SD))	0.10 (0.09)	0.11 (0.09)		0.09 (0.09)	0.001
Contextual risk domain (mean (SD))	0.16 (0.18)	0.20 (0.20)		0.25 (0.14)	<0.001
Personal risk domain (mean (SD))	0.03 (0.07)	0.05 (0.09)		0.11 (0.12)	<0.001
Interpersonal risk domain (mean (SD))	0.07 (0.09)	0.09 (0.13)		0.05 (0.09)	<0.001
Child sex, boys (*n* (%))	611 (49.9)	453 (47.7)		388 (49.1)	0.599
Gestational age at birth, weeks (mean (SD))	40.2 (1.5)	40.1 (1.4)		39.6 (1.5)	<0.001
Gestational weight at birth, grams (mean (SD))	3557 (506)	3529 (507)		3495 (479)	0.026
Maternal age at birth, years (mean (SD))	32.2 (4.2)	32.0 (4.3)		29.7 (4.4)	<0.001
Maternal tobacco smoking during pregnancy (*n* (%))†					
Never smoked during pregnancy	857 (70.0)	665 (70.1)	Never a smoker	481 (60.9)	
Smoked until pregnancy was known	109 (8.9)	78 (8.2)	Former smoker	219 (27.7)	
Continued smoking in pregnancy	159 (13.0)	146 (15.4)	Current smoker	90 (11.4)	
Maternal alcohol consumption during pregnancy (*n* (%))†					
Never drank during pregnancy	351 (28.7)	302 (31.8)	<1 Glass per week	>357 (>45.2)	
Drank until pregnancy was known	170 (13.9)	136 (14.3)	1+ Glass per week	170 (21.6)	
Continued drinking occasionally	551 (45.0)	381 (40.1)	1-2 Glasses per week	11 (1.4)	
Continued drinking frequently (1+ glass/week for 2+ trimesters)‡	152 (12.4)	130 (13.7)	>3 Glasses per week	<5 (<0.6)	

^*^Groups were compared using ANOVA testing.

^†^Maternal alcohol consumption and tobacco smoking was applied as continuous average score over two trimesters. Shown here as categorical for descriptive purposes.

^†^Where needed, approximate cell sizes are shown to ensure that exact cell sizes <5 (which may include zero) cannot be recovered from other information provided in this figure, in line with ALSPAC requirement.

### GxEmodel: SNP by prenatal stress interactions and DNA methylation

Five SNP-by-prenatal-stress interactions on DNAm were identified after Bonferroni correction, including five unique SNPs and two unique CpGs. Firstly, an association in *cis* of rs12901653 in *CHD2* in interaction with cumulative prenatal stress was found for DNAm at a nearby (64862 bp) cg24317086 (B = -0.026, SE = 0.003, *p* = 4.07×10^-16^), for which *CHASERR*, or *CHD2 Adjacent Suppressive Regulatory RNA* is the nearest gene. Secondly, *trans*-associations of 4 SNPs in or near *ORC5* in interaction with cumulative prenatal stress were found for DNAm at cg06592260, which is located in *EPPK1* (rs7642426: B = -0.013, SE = 0.002, *p* = 1.12×10^-13^; rs10279675: B = -0.012, SE = 0.002, *p* = 2.76×10^-13^; rs2188287: B = -0.013, SE = 0.002, *p* = 1.85×10^-13^; rs10251976: B = -0.012, SE = 0.002, *p* = 7.32×10^-13^). The adjusted R2 of the significant SNP-by-prenatal-stress interaction terms ranged between 0.02 and 0.03 and had a median (IQR) of 0.02 (0.02-0.02). As these SNPs were in high LD (>0.9 in all [sub-]cohorts), they were not independent (Supplemental Fig. 2). Results are depicted in Table 2 and Fig. 1. For all associations except the interaction of rs12901653 with cumulative prenatal stress on cg24317086, heterogeneity between (sub-)cohorts was low (I^2^ = 0.0). Rs12901653 showed considerable heterogeneity (I^2^ = 93.5), as associations for *GENR 450 K* and *GENR EPIC* were negative, whereas it was positive (although *p* > 0.05) for *ALSPAC 450 K* (forest plot in Supplemental Fig. 3).

**Table 2:** Genome- and epigenome-wide associations of SNP by cumulative prenatal stress interactions and DNA methylation.

SNP	SNP CHR:BP	CpG	CpG CHR:BP	B (SE)	*p*	adjusted R^2^*	direction†	I^2^	(nearest) gene SNP‡	(nearest) gene CpG‡	SNP-CpG distance (bp)
rs12901653	15:93488404	cg24317086	15:93423542	-0.0262 (0.0032)	4.067×10^-16^	0.03	--+	93.5	*CHD2*	*(CHASERR)*	64,862
rs76542426	7:103856777	cg06592260	8:144941878	-0.0128 (0.0017)	1.12×10^-13^	0.02	---	0.0	*(ORC5)*	*EPPK1*	41,085,101
rs10279675	7:103846781	cg06592260	8:144941878	-0.0123 (0.0017)	2.76×10^-13^	0.02	---	0.0	*ORC5*	*EPPK1*	41,095,097
rs2188287	7:103867883	cg06592260	8:144941878	-0.0126 (0.0017)	1.85×10^-13^	0.02	---	0.0	*(ORC5)*	*EPPK1*	41,073,995
rs10251976	7:103834690	cg06592260	8:144941878	-0.0124 (0.0017)	7.32×10^-13^	0.02	---	0.0	*ORC5*	*EPPK1*	41,107,188

^*^ Adjusted R [2] represents a sample-size weighted average over the (sub-)cohorts.

^†^ Direction indicates direction of estimate for *GENR 450 K*, *GENR EPIC*, and *ALSPAC 450 K*, respectively.

^‡^ For intergenic SNPs and CpGs the nearest gene is indicated in brackets.

**Fig. 1:**

Scatterplots of genome- and epigenome-wide associations of SNP-by-prenatal-stress interactions and DNA methylation.

### Gmodel: SNPs and DNA methylation

In the Gmodel, after Bonferroni correction, we found 691,202 associations between SNPs and DNAm, including 181,133 unique SNPs and 54,809 unique CpGs. As such, nearly half of all investigated SNPs (48%) could be marked as meQTLs, and more than half (59%) of the examined CpGs are under genetic control. In these results we find evidence of both polygenicity, i.e. multiple SNPs affecting the same CpG, as well as pleiotropy, i.e. the same SNP affecting multiple CpGs. Furthermore, 91% of SNP-CpG associations were in *cis*, 9% were in *trans* (distance of >1,000,000 bp; mean [SD] distance 136,731 [170,197] bp). The adjusted R2 of the significant prenatal stress terms ranged between 0.02 and 0.95 and had a median (IQR) of 0.04 (0.02-0.06). For 28% of associations, heterogeneity between (sub-)cohort results was considerable (I^2^ > 75).

### Emodel: Cumulative prenatal stress and DNA methylation

Three DNAm sites at birth were associated with exposure to cumulative prenatal stress after Bonferroni correction, including cg05575921 (B = -0.009, SE = 0.001, *p* = 3.81 × 10^-18^), located in *AHRR*, cg09935388 (B = -0.016, SE = 0.002, *p* = 2.79 × 10^-11^) in *GFI1*, and cg04180046 (B = -0.007, SE = 0.001, *p* = 6.73 × 10^-08^) in *MYO1G* (Table 3; Fig. 2). The adjusted R2 of the significant prenatal stress terms ranged between 0.02 and 0.03 and had a median (IQR) of 0.02 (0.01-0.02). For cg05575921, there was considerable heterogeneity between (sub-)cohort results (I^2^ = 78.1), for cg09935388 and cg04180046 no heterogeneity was detected (I^2^ = 0.0; forest plot in Supplemental Figure 4).

**Table 3:** Epigenome-wide associations of cumulative prenatal stress and DNA methylation.

CpG	CHR:BP	B (SE)	*p*	adjusted R^2^	direction	I^2^	gene
cg05575921	5:373378	-0.0086 (0.0010)	3.810×10^-18^	0.03	---	78.1	*AHRR*
cg09935388	1:92947588	-0.0161 (0.0024)	2.788×10^-11^	0.02	---	0.0	*GFI1*
cg04180046	7:45002736	0.0070 (0.0013)	6.732×10^-08^	0.02	+++	0.0	*MYO1G*

Direction indicates direction of estimate for *GENR 450 K*, *GENR EPIC*, and *ALSPAC 450 K*, respectively.

Adjusted R^2^ represents a sample-size weighted average over the (sub-)cohorts.

**Fig. 2:**

Scatterplots of epigenome-wide associations of cumulative prenatal stress and DNA methylation.

### Enrichment of cis- and trans- associations

Suggestive findings in the GxEmodel (3327 associations with 3248 unique SNPs and 2613 unique CpGs) and Gmodel (1,088,683 associations with 223,254 unique SNPs and 62,826 unique CpGs) were compared to test whether GxEmodel and Gmodel results differed in distance between associated SNP and CpGs. Among suggestive GxEmodel findings, only 1% was in cis (mean [SD] distance 17,767 [256,557] bp), whereas 89% of suggestive Gmodel was in cis (mean [SD] distance 155,089 [183,495] bp). This difference was significant (OR = 1498.2 [95% CI = 983.0-2915.3], p < 2.23×10^-308^).

### Enrichments of main effect model associations in GxEmodel

Suggestive SNPs and CpGs in the GxEmodel and Gmodel were compared to test whether a suggestive association in the Gmodel increased the chance of a suggestive association in the GxEmodel. This did not seem to be the case, as suggestive SNPs in the Gmodel were as likely to have been identified in the GxEmodel as other SNPs were (1 vs 1%; OR = 0.9 [95% CI = 0.9-1.0], *p* = 0.13). Similarly, suggestive CpGs in the Gmodel were as likely to be identified in the GxEmodel as other CpGs were (3 vs 3%; OR = 1.0 [95% CI = 0.9-1.1], *p* = 0.74). As a sensitivity analysis, we also checked for enrichment of SNPs and CpGs associated with meQTLs identified by others [34], and similarly found that suggestive SNPs and CpGs in the GxEmodel were as likely or even less likely to have been linked to an meQTL, whereas suggestive hits in the Gmodel were more likely to have previously been linked to an meQTL (Supplemental Results S1).

Furthermore, suggestive CpGs in the Emodel were as likely to have been identified as a suggestive CpG in the GxEmodel (7%) as other CpGs were (3%; OR = 2.5 [95% CI = 0.1-16.4], *p* = 0.35), although it should be noted that the number of suggestive findings for the Emodel was low with only 14 CpGs.

### CpG look-ups

From the GxEmodel, variation at cg24317086 has been previously associated with gestational age [40], age in childhood [27, 41], tissue type [42], Down syndrome [43], and C-reactive protein levels [44]. Variation at cg06592260 has been associated with age in childhood [27] and tissue type [42].

Full results for the lookup of the previously reported EWAS associations for the three CpGs found in the Emodel can be found in Supplemental Table 1. In brief, variation at cg05575921, cg09935388, and cg04180046 was related to maternal smoking during pregnancy with reported associations stemming from 6, 8 and 9 studies, respectively, and to smoking behavior (not in pregnancy) with reported associations in 27, 19, and 10 studies, respectively. Other associations were found, among others, for age in childhood [27], tissue type [42], alcohol consumption [45–47], maternal educational attainment during pregnancy [48], lung function [49–52] and post-traumatic stress syndrome [53, 54].

### Annotated gene look-up

The full results of the PheWASs are depicted in Supplemental Figures 5 to 10. In brief, genetic variants at *CHD2* were related to use of sun/UV protection, resting heart rate, free thyroxine levels, educational attainment, measures of body composition, uric acid levels, processed meat intake, pork intake, napping during the day, (standing) height, and schizophrenia. Genetic variants at *ORC5* have been related to risky behaviors, left and right entorhinal cortex volume, and drinking behavior. Genetic variants in or close to *EPPK1* (annotated to several CpGs of the significant GxEmodels) have been related to resting heart rate, skin tanning, body composition measures, and height.

Genetic variants at *AHRR* were related to skin colour, hair colour, male balding patterns, ulcerative colitis, hematocrit, hemoglobin, aspartate, fat measures, and height. Genetic variants annotated to *GFI1* were related to coronary artery disease, white blood cell measures, fat measures, multiple sclerosis, being a morning person, height, lung function, and asthma, eczema, and allergy related measures. Genetic variants at *MYO1G* were related to thyroid function, white blood cell measures, and height.

### Stress-domain-specific results

In the GxEmodel, none of the individual stress domains (life events, contextual risk, personal risk, interpersonal risk) provided a unique SNP-by-prenatal-stress contribution (*p* < 0.05) to the association with DNAm, over and above co-occurring domains (Supplemental Table 2). In the Emodel, contextual risk provided a unique contribution to DNAm at cg05575921 (*AHRR*, B = -0.008, SE = 0.001, *p* = 4.42x^-14^), cg09935388 (*GFI1*, B = -0.015, SE = 0.003, *p* = 6.18×10^-09^), and cg04180046 (*MYO1G*, B = 0.005, SE = 0.001, *p* = 7.36×10^-05^). In addition, interpersonal risk provided a unique contribution to cg05575921 (B = -0.003, SE = 0.001, *p* = 7.53×10^-03^) and cg09935388 (B = -0.007, SE = 0.003, *p* = 5.44×10^-03^). Life events and personal risk did not provide unique contributions in the significant Emodel associations (Supplemental Table 3).

### Mediation

In the GxEmodel, none of the significant SNP-by-prenatal-stress associations with DNAm were mediated by maternal tobacco smoking or alcohol consumption during pregnancy, gestational age, or birth weight. In the Emodel, all three cumulative prenatal stress associations with DNAm were mediated by maternal prenatal smoking (cg05575921: B_indirect_ = -0.007, 95% CI = -0.008;-0.006, *p* = 4.17×10^-60^; cg09935388: B_indirect_ = -0.010, 95% CI = -0.011;-0.008, *p* = 3.21×10^-30^; cg04180046: B_indirect_ = -0.006, 95% CI = -0.005;-0.007, *p* = 8.94×10^-40^), and not by any of the other mediators (Fig. 3).

**Fig. 3:**

Mediation of cumulative prenatal stress associations with DNA methylation by maternal tobacco smoking during pregnancy (prenatal smoking).

### Pathway enrichments

A Gene Ontology analysis of 3248 suggestive (*p* < 5×10^-08^) SNPs in the GxEmodel yielded 145 overrepresented pathways and 12 underrepresented pathways (Supplemental Table 4). The overrepresented pathways were predominantly linked to neuronal development and synaptic transmission. The underrepresented pathways were linked, amongst others, to DNA repair processes. A Gene Ontology analysis of 2613 suggestive CpGs in the GxEmodel yielded 35 overrepresented pathways (Supplemental Table 5), among which neuronal development-related pathways were predominant.

## Discussion

In this study, we investigated SNP-by-prenatal-stress interactions on DNAm at birth, for the first time at the genome- and epigenome-wide level. From the GxEmodel, we report five SNP-by-prenatal-stress interactions on DNAm after multiple testing correction, including five unique, of which two independent, SNPs in *CHD2* and *ORC5*, and two unique CpGs near *CHASERR* and in *EPPK1*. By comparison, the Gmodel yielded 691,202 associations of SNPs and DNAm, including 181,133 unique SNPs (48% of investigated SNPs) and 54,809 unique CpGs (59% of investigated CpGs), and the Emodel identified three associations between cumulative prenatal stress and DNAm at CpGs in *AHRR, GFI1*, and *MYO1G*, which are known DNAm loci for smoking exposure. Together, these results show that genetic main effects are by far stronger than prenatal stress effects alone or gene-by-prenatal stress interactions.

Significant results for the GxEmodel were scarce, which might in part be explained by the stringent Bonferroni multiple testing correction in which analyses were considered as independent. Due to the scale of the analyses, this resulted in a very low *p*-value threshold. However, a Bonferroni threshold might be overly stringent as there was a correlational structure among SNPs and among CpGs, and we also tested the same SNP 86,236 times, and the same CpG 374,152 times. Using a suggestive (genome-wide) significance threshold, Gene Ontology analyses pointed to enrichment of neuronal development related pathways. This may indicate that prenatal stress interacts with the child’s genome in many small ways to affect neuronal development, but that these associations only become visible with a less stringent threshold or a more powerful analysis. Moreover, previous GWASs have associated variation in *ORC5*, in or near which four of the associated SNPs were located with entorhinal cortex volume [55], an area important for memory processing, bordering the hippocampus and particularly rich in corticoid receptors [56]. These findings fit the developmental origins of health and disease perspective [57], which poses that the prenatal environment programs organ structure and function (in this case in interaction with the offspring genotype), as well as findings from our own lab that cumulative prenatal stress is related to childhood subcortical brain volumes [16], and that prenatal stress, *beyond* postnatal stress, predicts internalizing symptoms in childhood [15].

In contrast to the GxEmodel, significant associations in the Gmodel were abundant, indicating that many common SNPs are involved in epigenetic programming and in turn, that DNAm is under strong genetic control. This confirms earlier meQTL studies, which also identified numerous genetic effects on the epigenome [21, 34]. The abundance of genetic effects and far fewer GxE associations are contrary to the notion put forward by other studies that gene-environment interaction studies perform better than studies of genetic main effects alone in terms of predicting DNAm [12, 13, 58]. This may be explained by the different approaches to statistical inference used by these studies. In previous studies of genetic interaction effects with multiple prenatal environments [12, 13] and with adverse childhood experiences [58], G, E, and GxE models directly were compared for each CpG, using Akaike Information Criterion or R^2^, without adjustment for multiple testing. In contrast, we did not directly compare the different model fit per CpG. Instead, we compared the amount of Bonferroni-corrected significant results. It might therefore be possible that while GxE terms explain more DNAm variance than G- or E- main effect models do, these GxE effects are not large enough to survive the Bonferroni-correction applied here. However, Czamara et al. Czamara, Eraslan [13] also noted that ‘*GxE models appear to be winning by a significantly lager AIC margin over the next best model, when compared to the other types of winning models*’. If this had been the case for the current study, one might expect that the GxEmodel would have produced more hits than the Gmodel. Czamara, Eraslan [13] however, looked at a range of prenatal variables, so it may be that our findings are specific to prenatal stress.

What emerges from our results, however, is that the GxEmodel yields *different* results than when looking at genetic or environmental main effects alone. Follow-up analyses showed that SNPs and CpGs brought forward by the GxEmodel were not more likely to have been identified as, or related to an meQTL. For future GxE studies on DNAm, this means that only testing GxE interactions among significant findings in the Gmodel [12], would reduce the multiple testing burden, but might result in selective findings and may miss true GxE effects.

The Emodel yielded limited evidence of associations between cumulative prenatal stress and DNAm, which is in line with previous studies [9–11]. Moreover, whereas associations in the GxEmodel did not seem to be related to prenatal smoking and drinking behavior, gestational age or birthweight, the Emodel associations all could be largely explained by smoking behavior of the mother during pregnancy. Indeed, the look-up of related CpGs showed that these are top-hits in smoking EWASs [59–61]. Furthermore, whereas the GxEmodel results could not be explained by one of the types of stressors in particular, thereby ascribing to the notion that associations were due to the cumulative nature of prenatal stress rather than to the unique contribution of a specific stressor, contextual risk and interpersonal risk provided unique contributions to the results from the Emodel. These results fit with a recent EWAS meta-analysis of maternal educational attainment, often taken as an indicator of socio-economic position, which was also enriched for CpGs related to prenatal smoking [48] as well as an EWAS on victimization stress in children, of which results could also largely be explained by smoking [62]. It may be that prenatal stress in population-based samples does not provide enough variation to find true associations with DNAm at birth beyond those related to prenatal smoking, or simply that larger sample sizes are necessary to find small effect sizes. Alternatively, it may be that prenatal stress has limited direct effects on DNAm, but rather that its associations with DNAm are hidden in their dependency on genetic variation, meaning that interaction models are necessary to identify these associations. Our GxEmodel brought forward a similarly low amount of associations with DNAm, but potentially was burdened by a strict multiple testing correction. Again, larger sample sizes would be necessary to overcome the burden of multiple testing. Taken together, we conclude that our Emodel prenatal maternal stress associations with the offspring epigenome are not independent of maternal smoking behavior.

Results of this study should be interpreted in light of several limitations. First, effect sizes were small and Gene Ontology enrichment analysis of suggestive hits seemed to indicate that subthreshold findings are informative – hence larger sample sizes will likely be necessary to identify relevant gene-by-prenatal stress interactions with greater statistical power. Another method to improve statistical power would be to study a cohort with higher, or more variable stress levels, as the occurrence of prenatal stress was relatively low in the population-based samples used in the current study. However, to the best of our knowledge, this is the largest effort thus far to identify gene-by-prenatal stress effects on the epigenome. Moreover, we included a comprehensive measure of cumulative prenatal stress, capturing multiple domains of stress that often co-occur together – which has been uniquely harmonized between Generation R and ALSPAC, making it difficult to include a larger sample size at this time point. Second, there was heterogeneity between the (sub-)cohorts in the GxE association of a SNP in *CHD2* with a nearby CpG, which reduces the robustness of the finding. This finding was mainly driven by the results of the Generation R subcohorts, as the result in ALSPAC was nominally non-significant and even in the opposite direction. However, the finding was strong and consistent between the two Generation R subcohorts, that this still resulted in an overall significant finding. Moreover, as the I^2^ measure that was used for heterogeneity is relatively sensitive, this does not necessarily mean the finding is a false positive. Future studies are needed to examine this association in more detail, preferably in multiple cohorts. Third, whereas SNPs and CpGs included in these models span the full genome, we reduced the number of probes based on intercorrelation and/or variability to minimize the burden of multiple testing. This does mean, however, that it is possible that we missed associations. Fourth, since we took a genome-wide approach, including not only cis- but also trans-associations between SNPs and CpGs, we were computationally constrained and did not, as others [13, 58], include a G+Emodel with SNP and prenatal stress main effects. Given the weak evidence for associations in the Emodel, it is unlikely that a G+Emodel would have produced results too dissimilar from the Gmodel with only SNP main effects. Fifth, DNA methylation is tissue-specific and interactive effects of prenatal stress and offspring genotype on DNA methylation may differ between blood, which we used as an easily accessible tissue in population-based studies, and other, potentially more relevant tissues, such as brain – however, even in blood we found an epigenetic pattern of neurodevelopmental pathways. Sixth, the Generation R and ALSPAC are populations are generally selected towards being slightly healthier and more affluent than the general population, which may affect the generalizability of findings. It may also have reduced variation in prenatal stress and thereby the power to detect true associations. Also, as the epigenetic samples only include children of European ancestry, generalizability to populations of other ancestries may be limited. In the future, studies in populations of other ancestries are necessary to understand how genotype-by-prenatal-stress associates with DNAm at birth across different populations. Last, this is an observational study. Intervention studies on reducing prenatal stress [63–65] might help understand the degree to which gene-by-prenatal stress associations are causal in nature. Furthermore, more research would be necessary to understand the consequences of genotype-by-prenatal-stress associations found. The enrichment analyses indicated that neuronal development might be involved, yet more research is necessary to understand for which aspects of neuronal development this would be the case and to what degree.

In conclusion, in this comprehensive study of genotype-by-prenatal stress interactions on DNAm, we report suggestive findings that cumulative prenatal stress interacts with the child’s genome on DNA methylation in or close to genes related to neuronal development. Importantly, we found few significant associations in our environmental main effect model and our gene-by-prenatal stress interaction model, contrasting the many associations identified in our genetic main effect model. These results do not support the idea that gene-environment interactions on the epigenome are more abundant than gene effects alone, at least in the case of prenatal stress and when comparing the number of significant hits between different models. In the future, larger studies and studies including participants of different genetic ancestries are needed to identify associations with smaller effect sizes and generate results that are more generalizable.

## Supplementary information

SM Figure

SM Info

SM Results

SM Table 1

SM Table 2

SM Table 3

SM Table 4

SM Table 5

## Data Availability

The code that was produced to run the analyses is available at https://github.com/rosamulder/GxE-project.

## References

[1] Hentges RF, Graham SA, Plamondon A, Tough S, Madigan S. A developmental cascade from prenatal stress to child internalizing and externalizing problems. J Pediatr Psychol. 2019;44:1057–67. 31166597 10.1093/jpepsy/jsz044

[2] Dancause KN, Laplante DP, Hart KJ, O’Hara MW, Elgbeili G, Brunet A, et al. Prenatal stress due to a natural disaster predicts adiposity in childhood: the Iowa Flood Study. J Obes. 2015;2015:570541. 25874124 10.1155/2015/570541 PMC4383437

[3] Flanigan C, Sheikh A, DunnGalvin A, Brew BK, Almqvist C, Nwaru BI. Prenatal maternal psychosocial stress and offspring’s asthma and allergic disease: a systematic review and meta‐analysis. Clinical & Experimental Allergy. 2018;48:403–14. 29331049 10.1111/cea.13091

[4] Joubert BR, Felix JF, Yousefi P, Bakulski KM, Just AC, Breton C, et al. DNA methylation in newborns and maternal smoking in pregnancy: genome-wide consortium meta-analysis. The American Journal of Human Genetics. 2016;98:680–96. 27040690 10.1016/j.ajhg.2016.02.019 PMC4833289

[5] Mulder RH, Walton E, Neumann A, Houtepen LC, Felix JF, Bakermans-Kranenburg MJ, et al. Epigenomics of being bullied: changes in DNA methylation following bullying exposure. Epigenetics. 2020;15:750–64. 31992121 10.1080/15592294.2020.1719303 PMC7574379

[6] Vehmeijer FOL, Küpers LK, Sharp GC, Salas LA, Lent S, Jima DD, et al. DNA methylation and body mass index from birth to adolescence: meta-analyses of epigenome-wide association studies. Genome Med. 2020;12:1–15. 10.1186/s13073-020-00810-w PMC7687793 33239103

[7] Reese SE, Xu C-J, Herman T, Lee MK, Sikdar S, Ruiz-Arenas C, et al. Epigenome-wide meta-analysis of DNA methylation and childhood asthma. J Allergy Clin Immuno. 2019;143:2062–74. 10.1016/j.jaci.2018.11.043 PMC6556405 30579849

[8] Mulder RH, Rijlaarsdam J, Luijk MPCM, Verhulst FC, Felix JF, Tiemeier H, et al. Methylation matters: FK506 binding protein 51 (FKBP5) methylation moderates the associations of FKBP5 genotype and resistant attachment with stress regulation. Dev Psychopathol. 2017;29:491–503. 28401840 10.1017/S095457941700013X PMC5426551

[9] Sammallahti S, Hidalgo APC, Tuominen S, Malmberg A, Mulder RH, Brunst KJ, et al. Maternal anxiety during pregnancy and newborn epigenome-wide DNA methylation. Mol Psychiatry. 2021;26:1832–45. 33414500 10.1038/s41380-020-00976-0 PMC8595870

[10] Kotsakis Ruehlmann A, Sammallahti S, Cortés, Hidalgo AP, Bakulski KM, Binder EB, et al. Epigenome-wide meta-analysis of prenatal maternal stressful life events and newborn DNA methylation. Mol Psychiatry. 2023;28:5090–5100. 36899042 10.1038/s41380-023-02010-5 PMC12486153

[11] Rijlaarsdam J, Pappa I, Walton E, Bakermans-Kranenburg MJ, Mileva-Seitz VR, Rippe RCA, et al. An epigenome-wide association meta-analysis of prenatal maternal stress in neonates: A model approach for replication. Epigenetics. 2016;11:140–9. 26889969 10.1080/15592294.2016.1145329 PMC4846102

[12] Teh AL, Pan H, Chen L, Ong ML, Dogra S, Wong J, et al. The effect of genotype and in utero environment on interindividual variation in neonate DNA methylomes. Genome Res. 2014;24:1064–74. 24709820 10.1101/gr.171439.113 PMC4079963

[13] Czamara D, Eraslan G, Page CM, Lahti J, Lahti-Pulkkinen M, Hämäläinen E, et al. Integrated analysis of environmental and genetic influences on cord blood DNA methylation in new-borns. Nat Commun. 2019;10:1–18. 31186427 10.1038/s41467-019-10461-0 PMC6559955

[14] Cecil CAM, Lysenko LJ, Jaffee SR, Pingault JB, Smith RG, Relton CL, et al. Environmental risk, oxytocin receptor gene (OXTR) methylation and youth callous-unemotional traits: a 13-year longitudinal study. Mol Psychiatry. 2014;19:1071–7. 25199917 10.1038/mp.2014.95 PMC4231290

[15] Defina S, Woofenden T, Baltramonaityte V, Pariante CM, Lekadir K, Jaddoe VWV, et al. Effects of pre-and postnatal early-life stress on internalizing, adiposity and their comorbidity. J Am Acad Child Adolesc Psychiatry. 2024;63:255–65. 10.1016/j.jaac.2023.05.034 37453606

[16] Bolhuis K, Mulder RH, de Mol CL, Defina S, Warrier V, White T, et al. Mapping gene by early life stress interactions on child subcortical brain structures: A genome‐wide prospective study. JCPP Advances. 2022;2:e12113. 36777645 10.1002/jcv2.12113 PMC7614163

[17] Kooijman MN, Kruithof CJ, van Duijn CM, Duijts L, Franco OH, van IJzendoorn MH, et al. The Generation R Study: design and cohort update 2017. Eur J Epidemiol. 2016;31:1243–64. 28070760 10.1007/s10654-016-0224-9 PMC5233749

[18] Fraser A, Macdonald-Wallis C, Tilling K, Boyd A, Golding J, Davey Smith G, et al. Cohort profile: the avon longitudinal study of parents and children: ALSPAC mothers cohort. Int J Epidemiol. 2012;42:97–110. 22507742 10.1093/ije/dys066 PMC3600619

[19] Boyd A, Golding J, Macleod J, Lawlor DA, Fraser A, Henderson J, et al. Cohort profile: the ‘children of the 90s’—the index offspring of the avon longitudinal study of parents and children. Int J Epidemiol. 2013;42:111–27. 22507743 10.1093/ije/dys064 PMC3600618

[20] Medina-Gomez C, Felix JF, Estrada K, Peters MJ, Herrera L, Kruithof CJ, et al. Challenges in conducting genome-wide association studies in highly admixed multi-ethnic populations: the Generation R Study. Eur J Epidemiol. 2015;30:317–30. 25762173 10.1007/s10654-015-9998-4 PMC4385148

[21] Gaunt TR, Shihab HA, Hemani G, Min JL, Woodward G, Lyttleton O, et al. Systematic identification of genetic influences on methylation across the human life course. Genome Biol. 2016;17:61. 27036880 10.1186/s13059-016-0926-z PMC4818469

[22] Purcell S, Neale B, Todd-Brown K, Thomas L, Ferreira MAR, Bender D, et al. PLINK: a tool set for whole-genome association and population-based linkage analyses. The American journal of human genetics. 2007;81:559–75. 17701901 10.1086/519795 PMC1950838

[23] Buuren Sv, Groothuis-Oudshoorn K mice: Multivariate imputation by chained equations in R. *Journal of statistical software* 2010: 1-68.

[24] R Core Team. R: A language and environment for statistical computing. 2013.

[25] Lehne B, Drong AW, Loh M, Zhang W, Scott WR, Tan S-T, et al. A coherent approach for analysis of the Illumina HumanMethylation450 BeadChip improves data quality and performance in epigenome-wide association studies. Genome Biol. 2015;16:37. 25853392 10.1186/s13059-015-0600-x PMC4365767

[26] Min JL, Hemani G, Davey Smith G, Relton C, Suderman M, Hancock J. Meffil: efficient normalization and analysis of very large DNA methylation datasets. Bioinformatics. 2018;34:3983–9. 29931280 10.1093/bioinformatics/bty476 PMC6247925

[27] Mulder RH, Neumann A, Cecil CAM, Walton E, Houtepen LC, Simpkin AJ, et al. Epigenome-wide change and variation in DNA methylation in childhood: Trajectories from birth to late adolescence. Hum Mol Genet. 2021;30:119–34. 33450751 10.1093/hmg/ddaa280 PMC8033147

[28] Chen Y-a, Choufani S, Grafodatskaya D, Butcher DT, Ferreira JC, Weksberg R. Cross-reactive DNA microarray probes lead to false discovery of autosomal sex-associated DNA methylation. The American Journal of Human Genetics. 2012;91:762–4. 23040499 10.1016/j.ajhg.2012.06.020 PMC3484480

[29] Gervin K, Salas LA, Bakulski KM, Van Zelm MC, Koestler DC, Wiencke JK, et al. Systematic evaluation and validation of reference and library selection methods for deconvolution of cord blood DNA methylation data. Clin Epigenetics. 2019;11:1–15. 31455416 10.1186/s13148-019-0717-y PMC6712867

[30] Leek JT, Storey JD. Capturing heterogeneity in gene expression studies by surrogate variable analysis. PLoS Genet. 2007;3:e161. 17907809 10.1371/journal.pgen.0030161 PMC1994707

[31] Pan H, Holbrook JD, Karnani N, Kwoh CK. Gene, Environment and Methylation (GEM): a tool suite to efficiently navigate large scale epigenome wide association studies and integrate genotype and interaction between genotype and environment. BMC Bioinformatics. 2016;17:1–8. 27480116 10.1186/s12859-016-1161-z PMC4970299

[32] Willer CJ, Li Y, Abecasis GR. METAL: fast and efficient meta-analysis of genomewide association scans. Bioinformatics. 2010;26:2190–1. 20616382 10.1093/bioinformatics/btq340 PMC2922887

[33] Chandler J, Cumpston M, Li T, Page MJ, Welch V Cochrane handbook for systematic reviews of interventions. *Hoboken**:**Wiley* 2019.

[34] Min JL, Hemani G, Hannon E, Dekkers KF, Castillo-Fernandez J, Luijk R, et al. Genomic and phenotypic insights from an atlas of genetic effects on DNA methylation. Nat Genet. 2021;53:1311–21. 34493871 10.1038/s41588-021-00923-x PMC7612069

[35] Battram T, Yousefi P, Crawford G, Prince C, Babaei MS, Sharp G, et al. The EWAS Catalog: a database of epigenome-wide association studies. Wellcome Open Res. 2022;7:41. 35592546 10.12688/wellcomeopenres.17598.1 PMC9096146

[36] Liu X, Tian D, Li C, Tang B, Wang Z, Zhang R, et al. GWAS Atlas: an updated knowledgebase integrating more curated associations in plants and animals. Nucleic Acids Res. 2023;51:D969–76. 36263826 10.1093/nar/gkac924 PMC9825481

[37] Wang K, Li M, Hakonarson H. ANNOVAR: functional annotation of genetic variants from high-throughput sequencing data. Nucleic Acids Res. 2010;38:e164–e164. 20601685 10.1093/nar/gkq603 PMC2938201

[38] Rosseel Y. lavaan: An R package for structural equation modeling. J Stat Softw. 2012;48:1–36.

[39] Grote S, Grote MS Package ‘GOfuncR’. 2018.

[40] Bohlin J, Håberg SE, Magnus P, Reese SE, Gjessing HK, Magnus MC, et al. Prediction of gestational age based on genome-wide differentially methylated regions. Genome Biol. 2016;17:207. 27717397 10.1186/s13059-016-1063-4 PMC5054559

[41] Li C, Gao W, Gao Y, Yu C, Lv J, Lv R, et al. Age prediction of children and adolescents aged 6-17 years: an epigenome-wide analysis of DNA methylation. Aging. 2018;10:1015. 29754148 10.18632/aging.101445 PMC5990383

[42] Islam SA, Goodman SJ, MacIsaac JL, Obradović J, Barr RG, Boyce WT, et al. Integration of DNA methylation patterns and genetic variation in human pediatric tissues help inform EWAS design and interpretation. Epigenetics Chromatin. 2019;12:1–18. 30602389 10.1186/s13072-018-0245-6 PMC6314079

[43] Henneman P, Bouman A, Mul A, Knegt L, van der Kevie-Kersemaekers A-M, Zwaveling-Soonawala N, et al. Widespread domain-like perturbations of DNA methylation in whole blood of Down syndrome neonates. PLoS ONE. 2018;13:e0194938. 29601581 10.1371/journal.pone.0194938 PMC5877863

[44] Hillary RF, Ng HK, McCartney DL, Elliott HR, Walker RM, Campbell A, et al. Blood-based epigenome-wide analyses of chronic low-grade inflammation across diverse population cohorts. Cell Genomics. 2024;4:100544. 38692281 10.1016/j.xgen.2024.100544 PMC11099341

[45] Dugué PA, Wilson R, Lehne B, Jayasekara H, Wang X, Jung CH, et al. Alcohol consumption is associated with widespread changes in blood DNA methylation: analysis of cross‐sectional and longitudinal data. Addict Biol. 2021;26:e12855. 31789449 10.1111/adb.12855

[46] Liu C, Marioni RE, Hedman ÅK, Pfeiffer L, Tsai P-C, Reynolds LM, et al. A DNA methylation biomarker of alcohol consumption. Mol Psychiatry. 2018;23:422–33. 27843151 10.1038/mp.2016.192 PMC5575985

[47] Stephenson M, Bollepalli S, Cazaly E, Salvatore JE, Barr P, Rose RJ, et al. Associations of alcohol consumption with epigenome‐wide DNA methylation and epigenetic age acceleration: individual‐level and co‐twin comparison analyses. Alcoholism: Clinical and Experimental Research. 2021;45:318–28. 33277923 10.1111/acer.14528 PMC8120951

[48] Choudhary P, Monasso GS, Karhunen V, Ronkainen J, Mancano G, Howe CG, et al. Maternal educational attainment in pregnancy and epigenome-wide DNA methylation changes in the offspring from birth until adolescence. Mol Psychiatry. 2024;29:348–58. 38052982 10.1038/s41380-023-02331-5 PMC11116099

[49] Bermingham ML, Walker RM, Marioni RE, Morris SW, Rawlik K, Zeng Y, et al. Identification of novel differentially methylated sites with potential as clinical predictors of impaired respiratory function and COPD. EBioMedicine. 2019;43:576–86. 30935889 10.1016/j.ebiom.2019.03.072 PMC6557748

[50] Imboden M, Wielscher M, Rezwan FI, Amaral AFS, Schaffner E, Jeong A, et al. Epigenome-wide association study of lung function level and its change. European Respiratory Journal. 2019;54:1900457. 31073081 10.1183/13993003.00457-2019 PMC6610463

[51] Carmona JJ, Barfield RT, Panni T, Nwanaji-Enwerem JC, Just AC, Hutchinson JN, et al. Metastable DNA methylation sites associated with longitudinal lung function decline and aging in humans: an epigenome-wide study in the NAS and KORA cohorts. Epigenetics. 2018;13:1039–55. 30343628 10.1080/15592294.2018.1529849 PMC6342072

[52] Terzikhan N, Xu H, Edris A, Bracke KR, Verhamme FM, Stricker BHC, et al. Epigenome-wide association study on diffusing capacity of the lung. ERJ Open Res. 2021;7:00567–2020. 33748261 10.1183/23120541.00567-2020 PMC7957297

[53] Logue MW, Miller MW, Wolf EJ, Huber BR, Morrison FG, Zhou Z, et al. An epigenome-wide association study of posttraumatic stress disorder in US veterans implicates several new DNA methylation loci. Clin Epigenetics. 2020;12:1–14. 10.1186/s13148-020-0820-0 PMC7071645 32171335

[54] Smith AK, Ratanatharathorn A, Maihofer AX, Naviaux RK, Aiello AE, Amstadter AB, et al. Epigenome-wide meta-analysis of PTSD across 10 military and civilian cohorts identifies methylation changes in AHRR. Nat Commun. 2020;11:5965. 33235198 10.1038/s41467-020-19615-x PMC7686485

[55] Zhao B, Luo T, Li T, Li Y, Zhang J, Shan Y, et al. Genome-wide association analysis of 19,629 individuals identifies variants influencing regional brain volumes and refines their genetic co-architecture with cognitive and mental health traits. Nat Genet. 2019;51:1637–44. 31676860 10.1038/s41588-019-0516-6 PMC6858580

[56] Lupien SJ, Maheu F, Tu M, Fiocco A, Schramek TE. The effects of stress and stress hormones on human cognition: Implications for the field of brain and cognition. Brain Cogn. 2007;65:209–37. 17466428 10.1016/j.bandc.2007.02.007

[57] Barker DJ. The fetal and infant origins of adult disease. BMJ: British Medical Journal. 1990;301:1111. 2252919 10.1136/bmj.301.6761.1111 PMC1664286

[58] Czamara D, Tissink E, Tuhkanen J, Martins J, Awaloff Y, Drake AJ, et al. Combined effects of genotype and childhood adversity shape variability of DNA methylation across age. Transl Psychiatry. 2021;11:88. 33526782 10.1038/s41398-020-01147-z PMC7851167

[59] Andersen AM, Philibert RA, Gibbons FX, Simons RL, Long J. Accuracy and utility of an epigenetic biomarker for smoking in populations with varying rates of false self‐report. American Journal of Medical Genetics Part B: Neuropsychiatric Genetics. 2017;174:641–50. 10.1002/ajmg.b.32555 PMC5653254 28816414

[60] Philibert R, Hollenbeck N, Andersen E, McElroy S, Wilson S, Vercande K, et al. Reversion of AHRR demethylation is a quantitative biomarker of smoking cessation. Front Psychiatry. 2016;7:55. 27092088 10.3389/fpsyt.2016.00055 PMC4822186

[61] Bojesen SE, Timpson N, Relton C, Smith GD, Nordestgaard BG. AHRR (cg05575921) hypomethylation marks smoking behaviour, morbidity and mortality. Thorax. 2017;72:646–53. 28100713 10.1136/thoraxjnl-2016-208789 PMC5520281

[62] Marzi SJ, Sugden K, Arseneault L, Belsky DW, Burrage J, Corcoran DL, et al. Analysis of DNA methylation in young people: limited evidence for an association between victimization stress and epigenetic variation in blood. American Journal of Psychiatry. 2018;175:517–29. 29325449 10.1176/appi.ajp.2017.17060693 PMC5988939

[63] Virgili M. Mindfulness-based interventions reduce psychological distress in working adults: a meta-analysis of intervention studies. Mindfulness. 2015;6:326–37.

[64] Turner K, McCarthy VL. Stress and anxiety among nursing students: A review of intervention strategies in literature between 2009 and 2015. Nurse Educ Pract. 2017;22:21–9. 27889624 10.1016/j.nepr.2016.11.002

[65] Yusufov M, Nicoloro-SantaBarbara J, Grey NE, Moyer A, Lobel M. Meta-analytic evaluation of stress reduction interventions for undergraduate and graduate students. Int J Stress Manag. 2019;26:132.

