# Supplementary material for: Interactive effects of genotype with prenatal stress on DNA methylation at birth
Source: Mol Psychiatry. 2025 Oct 24;30(12):5749–59. doi: 10.1038/s41380-025-03312-6 (PMC12602324; doi:10.1038/s41380-025-03312-6)
Supplement: Supplementary file 1 — SM Figure [file 41380_2025_3312_MOESM1_ESM.pdf]

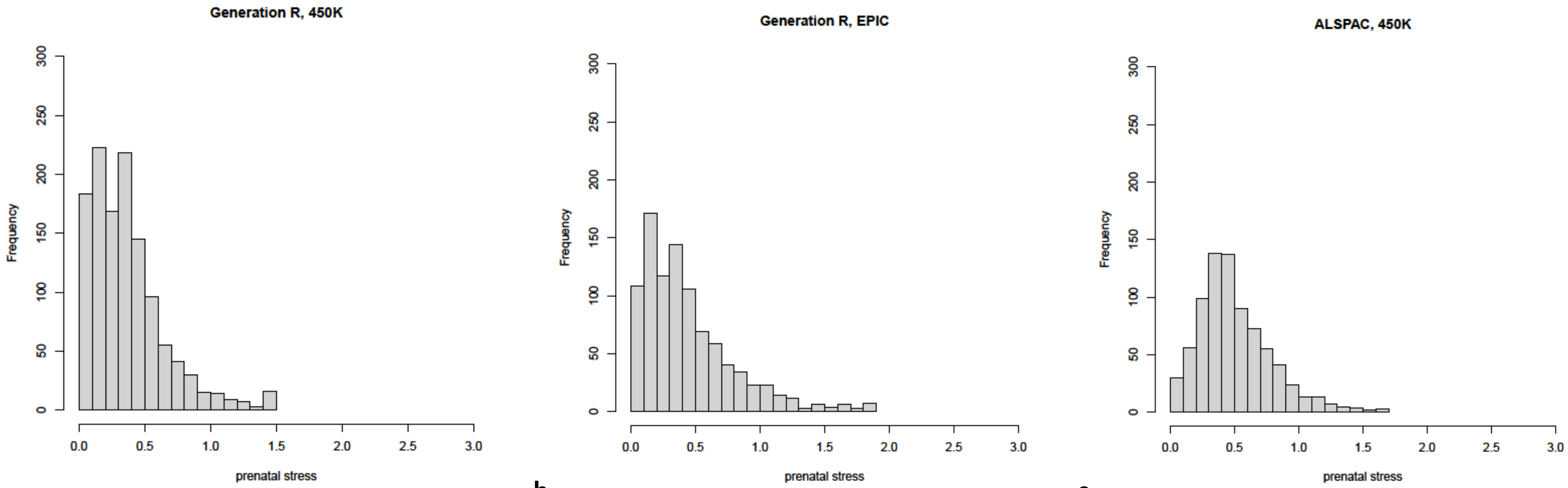


**a.** **b.** **c.**

**Supplemental Figure 1.** Histograms of prenatal maternal stress in the **a)** *GENR 450K* subcohort, **b)** *GENR EPIC* subcohort, and **c)** *ALSPAC 450K* cohort.

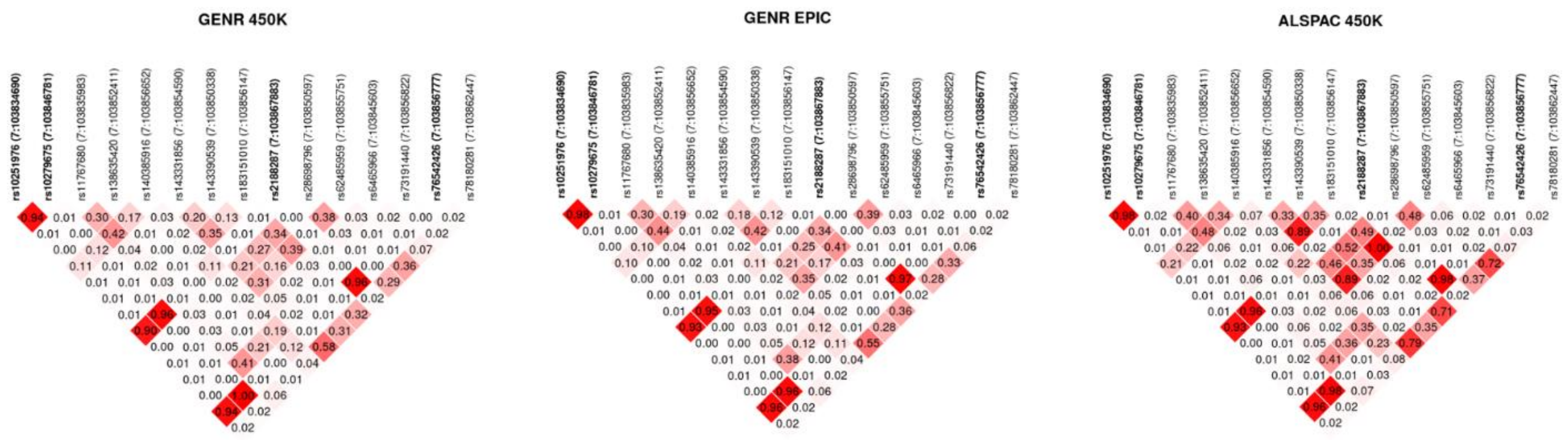


**Supplemental Figure 2.** Linkage disequilibrium structure of significant (in bold) and nearby GxEmodel SNPs on chromosome 7.

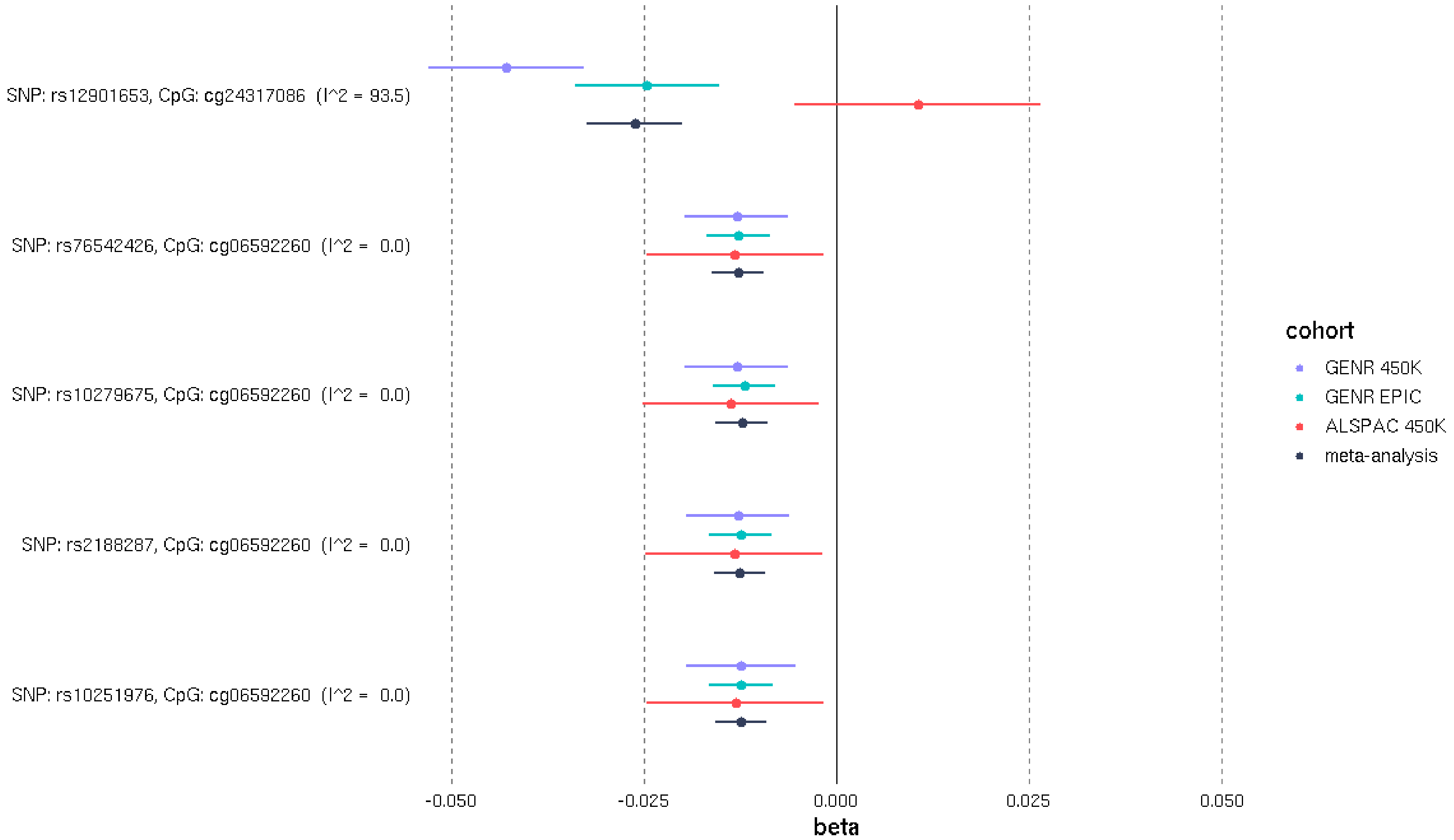


**Supplemental Figure 3.** Forest plot for significant (*p*<1.55x10$^{-12}$) GxEmodel results of SNP by cumulative prenatal stress interaction associations with DNA methylation

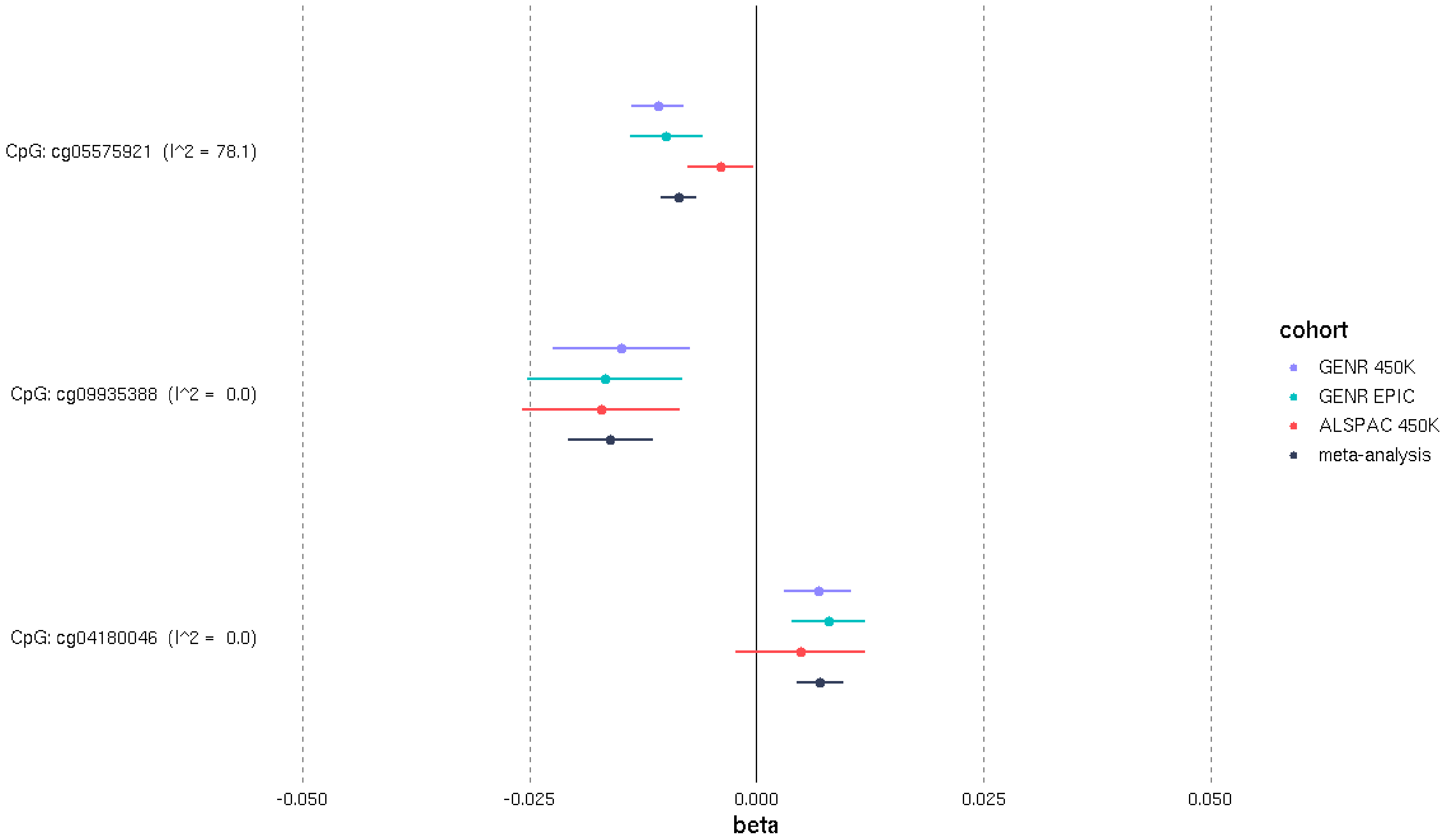


**Supplemental Figure 4.** Forest plot for significant (*p*<5.80x10$^{-07}$) Emodel results of cumulative prenatal stress associations with DNA methylation

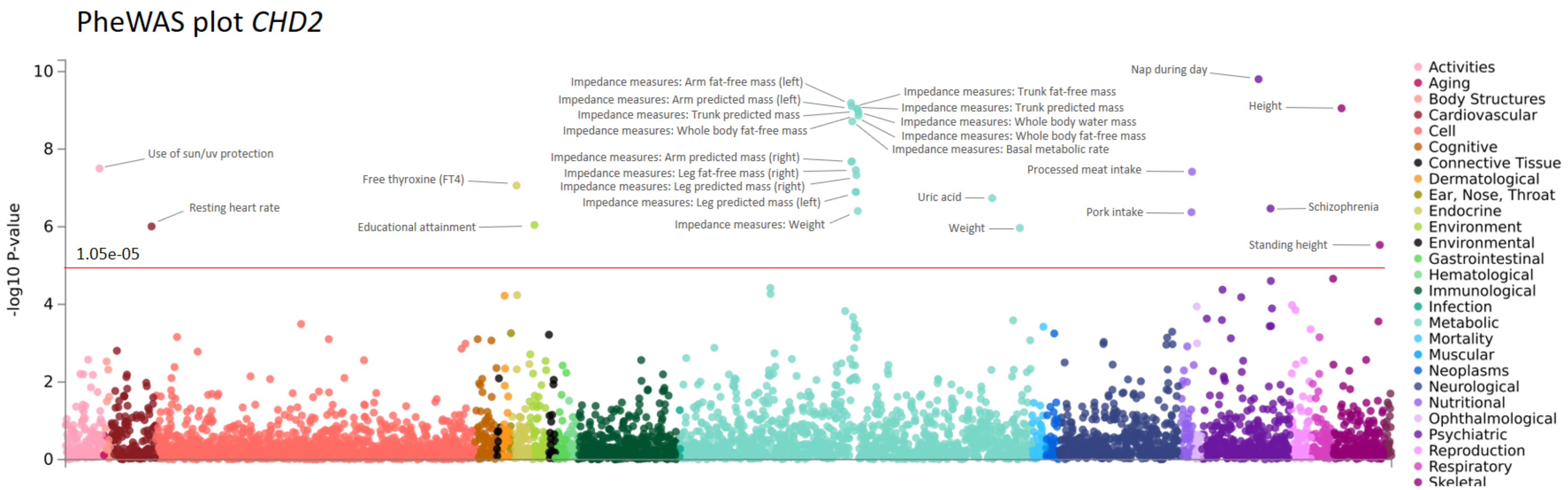


**Supplemental Figure 5.** Phenome-wide association study for *CHD2*, including 4756 GWASs, performed with the GWAS Atlas (https://atlas.ctglab.nl/PheWAS)

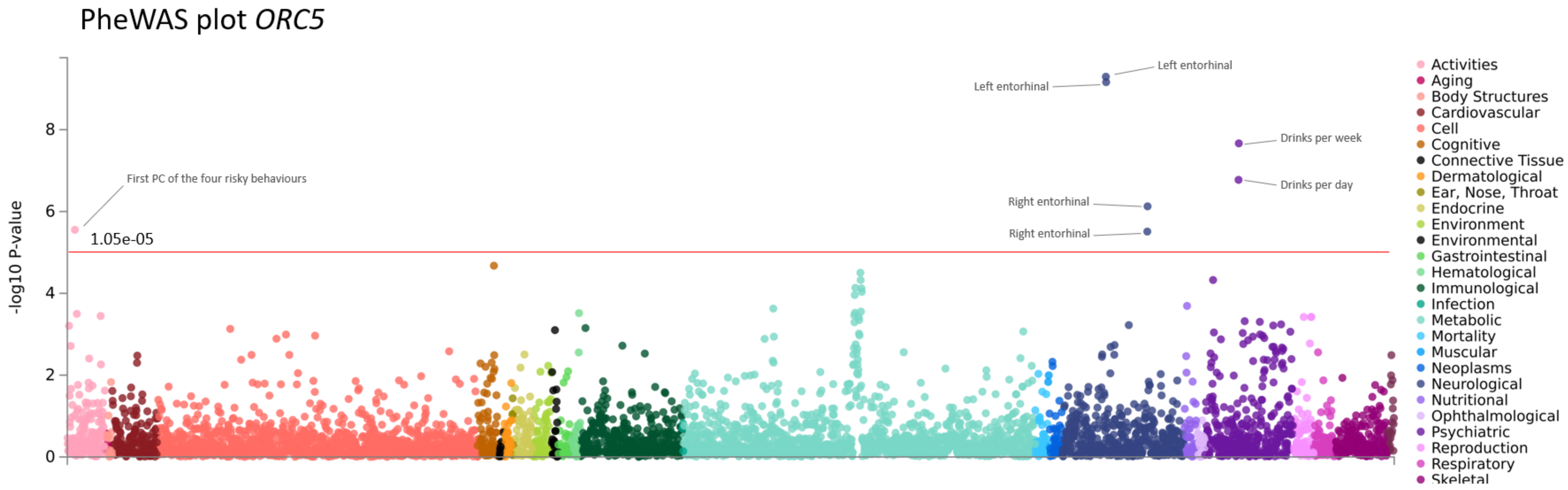


**Supplemental Figure 6.** Phenome-wide association study for *ORC5*, including 4756 GWASs, performed with the GWAS Atlas (https://atlas.ctglab.nl/PheWAS)

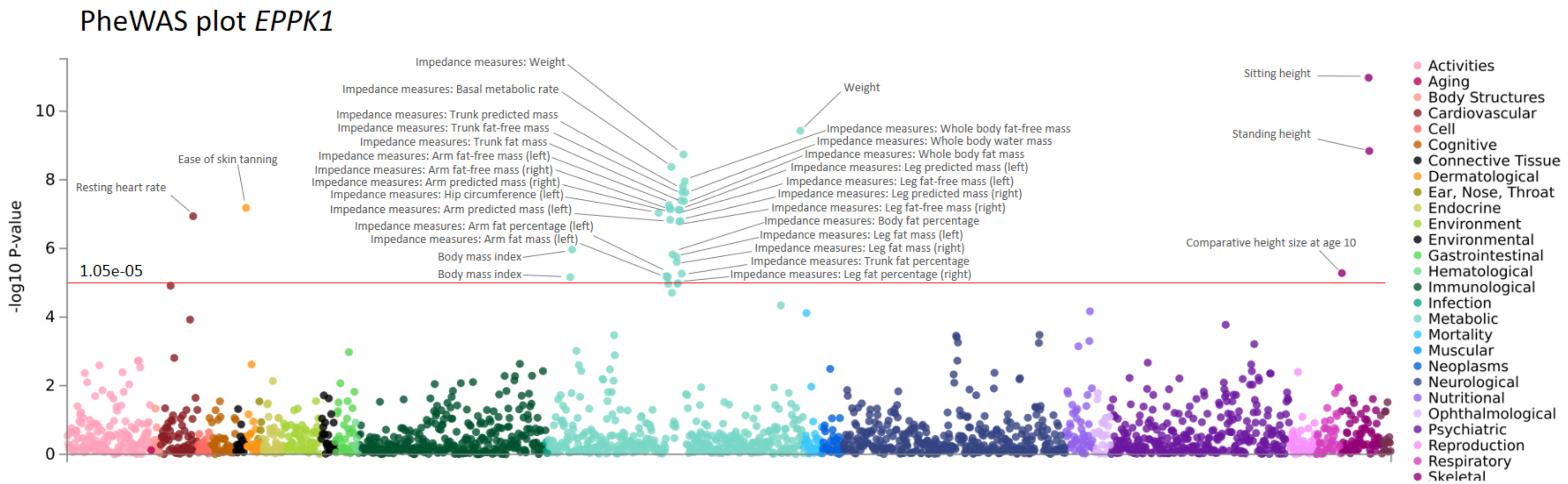


**Supplemental Figure 7.** Phenome-wide association study for *EPPK1*, including 4756 GWASs, performed with the GWAS Atlas (https://atlas.ctglab.nl/PheWAS)

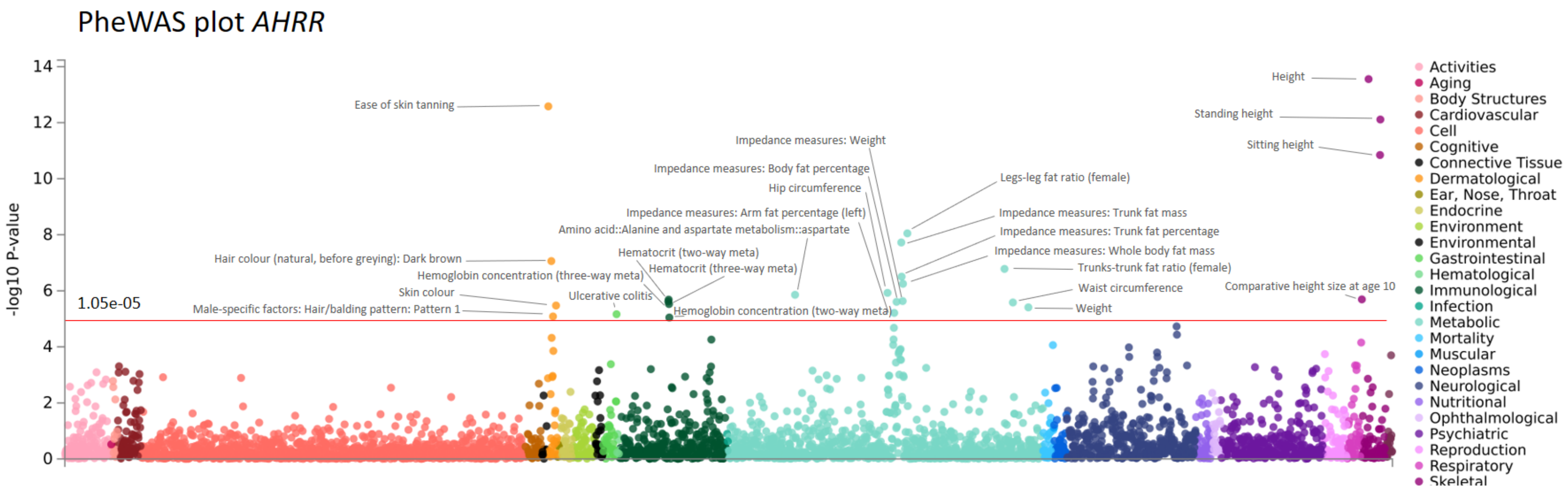


**Supplemental Figure 8.** Phenome-wide association study for *AHRR*, including 4756 GWASs, performed with the GWAS Atlas (https://atlas.ctglab.nl/PheWAS)

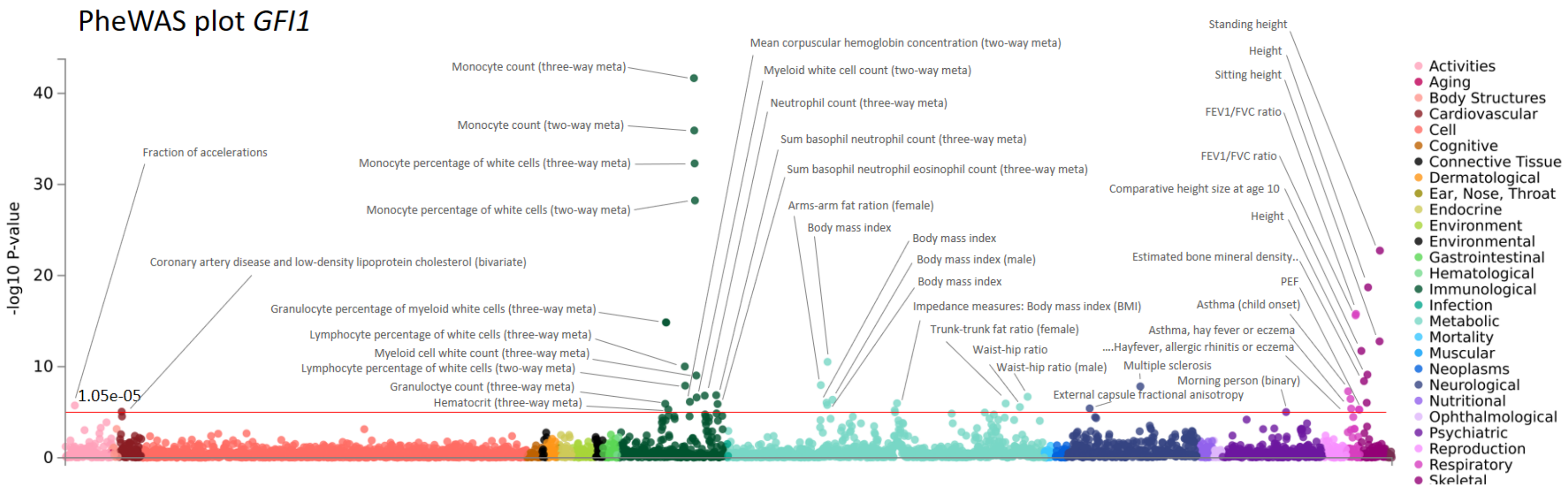


**Supplemental Figure 9.** Phenome-wide association study for *GFI1*, including 4756 GWASs, performed with the GWAS Atlas (https://atlas.ctglab.nl/PheWAS)

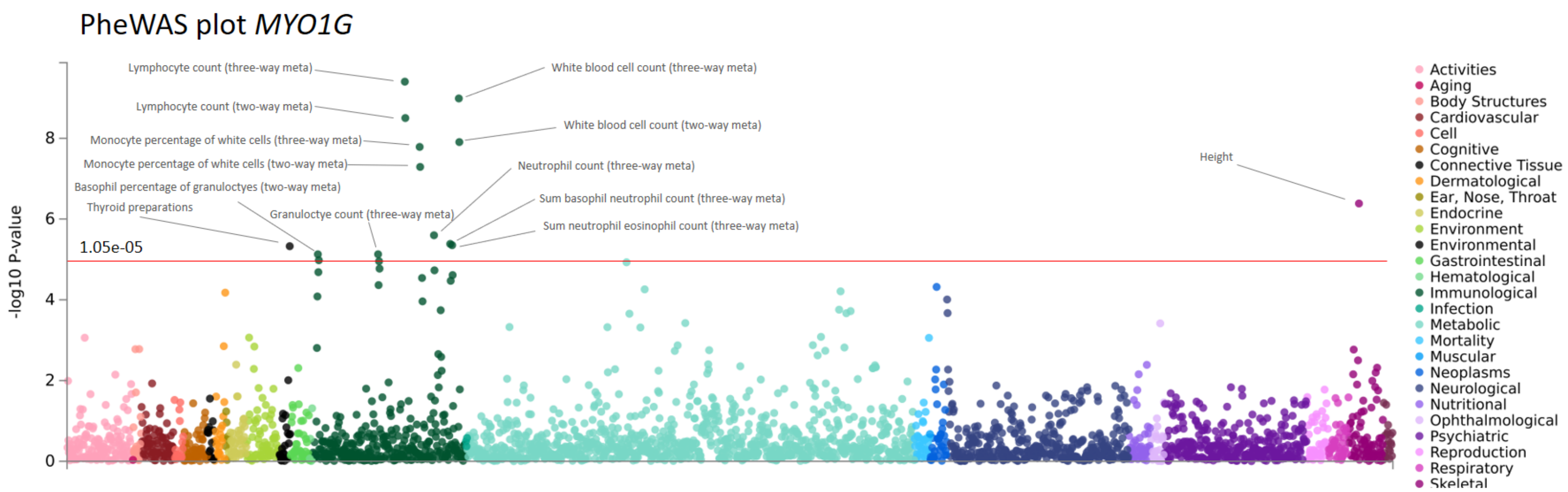


**Supplemental Figure 10.** Phenome-wide association study for *MYO1G*, including 4756 GWASs, performed with the GWAS Atlas (https://atlas.ctglab.nl/PheWAS)
