# Supplementary material for: Interactive effects of genotype with prenatal stress on DNA methylation at birth
Source: Mol Psychiatry. 2025 Oct 24;30(12):5749–59. doi: 10.1038/s41380-025-03312-6 (PMC12602324; doi:10.1038/s41380-025-03312-6)
Supplement: Supplementary file 4 — SM Table 1 [file 41380_2025_3312_MOESM4_ESM.pdf]

Supplemental Table 1. EWAS Catalog look-ups of Emodel findings

CpG	Phenotype	Reference
cg05575921	Maternal smoking during pregnancy	Hannon et al., 2019; Joubert et al., 2016; Joubert et al., 2012; Markunas et al., 2014; Richmond et al., 2014; Sikdar et al., 2019
	Smoking	Ambatipudi et al., 2016; Barcelona et al., 2019; Chatziioannou et al., 2017; Christiansen et al., 2021; Dogan, Beach, & Philibert, 2017; Domingo-Relloso et al., 2020; Dugué et al., 2020; Guida et al., 2015; Joehanes et al., 2016; Marzi et al., 2018; Monick et al., 2012; Philibert, Beach, & Brody, 2012; Richmond et al., 2021; Sayols-Baixeras et al., 2015; Shah et al., 2014; Shenker et al., 2013; Sikdar et al., 2019; Sun et al., 2021; Terzikhan et al., 2021; Teschendorff et al., 2015; Tsaprouni et al., 2014; Wiklund et al., 2019; Xu et al., 2021; Zaghlool et al., 2015; Zeilinger et al., 2013; Zhang, Florath, Saum, & Brenner, 2016; Zhu et al., 2016
	Age in childhood	Mulder et al., 2021
	Age in adulthood	Hannum et al., 2013
	Sex	Singmann et al., 2015
	Tissue type	Islam et al., 2019
	Immunoglobulin G glycosylation	Wahl et al., 2018
	C-reactive protein levels	Hillary et al., 2024; Ligthart et al., 2016
	Lung function	Bermingham et al., 2019; Carmona et al., 2018; Imboden et al., 2019; Terzikhan et al., 2021
	Chronic obstructive pulmonary disease	Hillary et al., 2024
	Lung cancer	Fasanelli et al., 2015; Hillary et al., 2024; Sun et al., 2021; Zhao et al., 2022
	Ischaemic heart disease	Hillary et al., 2024
	Stroke	Hillary et al., 2024
	Actue myocardial infection	Fernández-Sanlés et al., 2021
	Carotid intima-media thickness	Portilla-Fernández et al., 2021
	Statin use	Ochoa-Rosales et al., 2020
	Body mass index	Demerath et al., 2015
	Waist circumference	Demerath et al., 2015
	Rheumatoid arthritis	Hillary et al., 2024
	Chronic pain	Hillary et al., 2024
	Incident liver cirrhosis	Hillary et al., 2024
	Chronic kidney disease	Hillary et al., 2024
	All-cause mortality	Colicino et al., 2020
	Aggressive behavior	van Dongen et al., 2021
	Educational attainment	Karlsson Linnér et al., 2017; van Dongen et al., 2018
	Maternal educational attainment during pregnancy	Choudhary et al., 2024
	Vinylphenol-sulfate metabolite levels	Petersen et al., 2014
	Cognitive abilities	Marioni et al., 2018
	Alcohol consumption	Dugué et al., 2021; Liu et al., 2018; Stephenson et al., 2021
	Coffee and tea consumption	Karabegović et al., 2021
	Diet quality	Do et al., 2021
	Birth weight	Küpers et al., 2019
	Post-traumatic stress disorder	Logue et al., 2020; Smith et al., 2011

	Series of protein levels	Gadd et al., 2022
cg09935388	Maternal smoking during pregnancy	Hannon et al., 2019; Joubert et al., 2016; Joubert et al., 2012; Markunas et al., 2014; Richmond et al., 2014; Sikdar et al., 2019; Wiklund et al., 2019; Xu et al., 2021
	Smoking	Ambatipudi et al., 2016; Barcelona et al., 2019; Besingi & Johansson, 2014; Chatziioannou et al., 2017; Christiansen et al., 2021; Dogan et al., 2017; Domingo-Relloso et al., 2020; Dugué et al., 2020; Guida et al., 2015; Joehanes et al., 2016; Marzi et al., 2018; Shenker et al., 2013; Sikdar et al., 2019; Sun et al., 2021; Teschendorff et al., 2015; Tsaprouni et al., 2014; Zeilinger et al., 2013; Zhang et al., 2016; Zhu et al., 2016
	Age in childhood	Mulder et al., 2021
	Fetal versus adult cells	Bonder et al., 2014
	Sex	Mulder et al., 2021
	Tissue type	Islam et al., 2019
	C-reactive protein levels	Hillary et al., 2024
	Lung function	Imboden et al., 2019
	Chronic obstructive pulmonary disease	Hillary et al., 2024
	Lung cancer	Hillary et al., 2024; Sun et al., 2021
	Ischaemic heart disease	Hillary et al., 2024
	Stroke	Hillary et al., 2024
	Body mass index	Demerath et al., 2015
	Waist circumference	Demerath et al., 2015
	Osteoarthritis	Hillary et al., 2024
	Chronic pain	Hillary et al., 2024
	Chronic kidney disease	Hillary et al., 2024
	Renal cell carcinoma	Wozniak et al., 2013
	Aggressive behavior	van Dongen et al., 2021
	Educational attainment	Karlsson Linnér et al., 2017; van Dongen et al., 2018
	Maternal educational attainment during pregnancy	Choudhary et al., 2024
	Vinylphenol-sulfate metabolite levels	Petersen et al., 2014
	Cognitive abilities	Marioni et al., 2018
	Alcohol consumption	Dugué et al., 2021; Liu et al., 2018
	Coffee and tea consumption	Karabegović et al., 2021
	Birth weight	Küpers et al., 2019
cg04180046	Maternal smoking during pregnancy	Hannon et al., 2019; Joubert et al., 2016; Joubert et al., 2012; Lee et al., 2015; Markunas et al., 2014; Richmond et al., 2014; Rzehak et al., 2016; Sikdar et al., 2019; Wiklund et al., 2019
	Smoking	Ambatipudi et al., 2016; Chatziioannou et al., 2017; Christiansen et al., 2021; Domingo-Relloso et al., 2020; P.-A. Dugué et al., 2020; Joehanes et al., 2016; Sikdar et al., 2019; Sun et al., 2021; Zeilinger et al., 2013; Zhu et al., 2016
	Childhood age	Mulder et al., 2021
	Tissue type	Islam et al., 2019
	Chronic obstructive pulmonary disease	Hillary et al., 2024
	Lung cancer	Hillary et al., 2024
	Ischaemic heart disease	Hillary et al., 2024
	Type 2 diabetes	Hillary et al., 2024

Chronic pain	Hillary et al., 2024
Aggressive behavior	van Dongen et al., 2021
Educational attainment	Karlsson Linnér et al., 2017; van Dongen et al., 2018
Maternal educational attainment during pregnancy	Choudhary et al., 2024
Fetal alcohol spectrum disorder	Portales-Casamar et al., 2016

References

Ambatipudi, S., Cuenin, C., Hernandez-Vargas, H., Ghantous, A., Le Calvez-Kelm, F., Kaaks, R., . . . Trichopoulou, A. (2016). Tobacco smoking-associated genome-wide DNA methylation changes in the EPIC study. Epigenomics, 8(5), 599-618.

Barcelona, V., Huang, Y., Brown, K., Liu, J., Zhao, W., Yu, M., . . . Sun, Y. V. (2019). Novel DNA methylation sites associated with cigarette smoking among African Americans. Epigenetics, 14(4), 383-391.

Bermingham, M. L., Walker, R. M., Marioni, R. E., Morris, S. W., Rawlik, K., Zeng, Y., . . . Adams, M. J. (2019). Identification of novel differentially methylated sites with potential as clinical predictors of impaired respiratory function and COPD. EBioMedicine, 43, 576-586.

Besingi, W., & Johansson, Å. (2014). Smoke-related DNA methylation changes in the etiology of human disease. Human molecular genetics, 23(9), 2290-2297.

Bonder, M. J., Kasela, S., Kals, M., Tamm, R., Lokk, K., Barragan, I., . . . Ivanov, M. (2014). Genetic and epigenetic regulation of gene expression in fetal and adult human livers. BMC Genomics, 15, 1-13.

Carmona, J. J., Barfield, R. T., Panni, T., Nwanaji-Enwerem, J. C., Just, A. C., Hutchinson, J. N., . . . Kunze, S. (2018). Metastable DNA methylation sites associated with longitudinal lung function decline and aging in humans: an epigenome-wide study in the NAS and KORA cohorts. Epigenetics, 13(10-11), 1039-1055.

Chatziioannou, A., Georgiadis, P., Hebels, D. G., Liampa, I., Valavanis, I., Bergdahl, I. A., . . . Siskos, A. P. (2017). Blood-based omic profiling supports female susceptibility to tobacco smoke-induced cardiovascular diseases. Scientific reports, 7, 42870.

Choudhary, P., Monasso, G. S., Karhunen, V., Ronkainen, J., Mancano, G., Howe, C. G., . . . Dou, J. (2024). Maternal educational attainment in pregnancy and epigenome-wide DNA methylation changes in the offspring from birth until adolescence. Mol. Psychiatr., 29(2), 348-358.

Christiansen, C., Castillo-Fernandez, J. E., Domingo-Relloso, A., Zhao, W., El-Sayed Moustafa, J. S., Tsai, P. C., . . . Kardia, S. L. R. (2021). Novel DNA methylation signatures of tobacco smoking with trans-ethnic effects. Clinical epigenetics, 13, 1-13.

Colicino, E., Marioni, R., Ward-Caviness, C., Gondalia, R., Guan, W., Chen, B., . . . Golareh, A. (2020). Blood DNA methylation sites predict death risk in a longitudinal study of 12,300 individuals. Aging (Albany NY), 12(14), 14092.

Demerath, E. W., Guan, W., Grove, M. L., Aslibekyan, S., Mendelson, M., Zhou, Y.-H., . . . Irvin, M. R. (2015). Epigenome-wide association study (EWAS) of BMI, BMI change and waist circumference in African American adults identifies multiple replicated loci. Human molecular genetics, 24(15), 4464-4479.

Do, W. L., Whitsel, E. A., Costeira, R., Masachs, O. M., Le Roy, C. I., Bell, J. T., . . . Horvath, S. (2021). Epigenome-wide association study of diet quality in the Women's Health Initiative and TwinsUK cohort. Int. J. Epidemiol., 50(2), 675-684.

Dogan, M. V., Beach, S. R. H., & Philibert, R. A. (2017). Genetically contextual effects of smoking on genome wide DNA methylation. American Journal of Medical Genetics Part B: Neuropsychiatric Genetics, 174(6), 595-607.

Domingo-Relloso, A., Riffo-Campos, A. L., Haack, K., Rentero-Garrido, P., Ladd-Acosta, C., Fallin, D. M., . . . Bozack, A. K. (2020). Cadmium, smoking, and human blood DNA methylation profiles in adults from the strong heart study. Environmental health perspectives, 128(6), 067005.

Dugué, P.-A., Jung, C.-H., Joo, J. E., Wang, X., Wong, E. M., Makalic, E., . . . Southey, M. C. (2020). Smoking and blood DNA methylation: an epigenome-wide association study and assessment of reversibility. Epigenetics, 15(4), 358-368.

Dugué, P.-A., Wilson, R., Lehne, B., Jayasekara, H., Wang, X., Jung, C. H., . . . Baglietto, L. (2021). Alcohol consumption is associated with widespread changes in blood DNA methylation: analysis of cross-sectional and longitudinal data. Addict. Biol., 26(1), e12855.

Fasanelli, F., Baglietto, L., Ponzi, E., Guida, F., Campanella, G., Johansson, M., . . . Naccarati, A. (2015). Hypomethylation of smoking-related genes is associated with future lung cancer in four prospective cohorts. Nat. Commun., 6(1), 10192.

Fernández-Sanlés, A., Sayols-Baixeras, S., Subirana, I., Sentí, M., Pérez-Fernández, S., de Castro Moura, M., . . . Elosua, R. (2021). DNA methylation biomarkers of myocardial infarction and cardiovascular disease. Clinical epigenetics, 13, 1-11.

Gadd, D. A., Hillary, R. F., McCartney, D. L., Shi, L., Stolicyn, A., Robertson, N. A., . . . Xueyi, S. (2022). Integrated methylome and phenome study of the circulating proteome reveals markers pertinent to brain health. Nat. Commun., 13(1), 4670.

Guida, F., Sandanger, T. M., Castagné, R., Campanella, G., Polidoro, S., Palli, D., . . . Panico, S. (2015). Dynamics of smoking-induced genome-wide methylation changes with time since smoking cessation. Human molecular genetics, 24(8), 2349-2359.

Hannon, E., Schendel, D., Ladd-Acosta, C., Grove, J., Hansen, C. S., Hougaard, D. M., . . . Bækvad-Hansen, M. (2019). Variable DNA methylation in neonates mediates the association between prenatal smoking and birth weight. Philosophical Transactions of the Royal Society B, 374(1770), 20180120.

Hannum, G., Guinney, J., Zhao, L., Zhang, L., Hughes, G., Sadda, S., . . . Gao, Y. (2013). Genome-wide methylation profiles reveal quantitative views of human aging rates. Molecular cell, 49(2), 359-367.

Hillary, R. F., Ng, H. K., McCartney, D. L., Elliott, H. R., Walker, R. M., Campbell, A., . . . Sattar, N. (2024). Blood-based epigenome-wide analyses of chronic low-grade inflammation across diverse population cohorts. Cell Genomics, 4(5).

Imboden, M., Wielscher, M., Rezwan, F. I., Amaral, A. F. S., Schaffner, E., Jeong, A., . . . Deary, I. J. (2019). Epigenome-wide association study of lung function level and its change. European Respiratory Journal, 54(1).

Islam, S. A., Goodman, S. J., MacIsaac, J. L., Obradović, J., Barr, R. G., Boyce, W. T., & Kobor, M. S. (2019). Integration of DNA methylation patterns and genetic variation in human pediatric tissues help inform EWAS design and interpretation. Epigenetics & chromatin, 12, 1-18.

Joehanes, R., Just, A. C., Marioni, R. E., Pilling, L. C., Reynolds, L. M., Mandaviya, P. R., . . . Aslibekyan, S. (2016). Epigenetic signatures of cigarette smoking. Circulation: Cardiovascular Genetics, 9(5), 436-447.

Joubert, B. R., Felix, J. F., Yousefi, P., Bakulski, K. M., Just, A. C., Breton, C., . . . Xu, C.-J. (2016). DNA methylation in newborns and maternal smoking in pregnancy: genome-wide consortium meta-analysis. The American Journal of Human Genetics, 98(4), 680-696.

Joubert, B. R., Håberg, S. E., Nilsen, R. M., Wang, X., Vollset, S. E., Murphy, S. K., . . . Cupul-Uicab, L. A. (2012). 450K epigenome-wide scan identifies differential DNA methylation in newborns related to maternal smoking during pregnancy. Environmental health perspectives, 120(10), 1425-1431.

Karabegović, I., Portilla-Fernandez, E., Li, Y., Ma, J., Maas, S. C. E., Sun, D., . . . Ambatipudi, S. (2021). Epigenome-wide association meta-analysis of DNA methylation with coffee and tea consumption. Nat. Commun., 12(1), 2830.

Karlsson Linnér, R., Marioni, R. E., Rietveld, C. A., Simpkin, A. J., Davies, N. M., Watanabe, K., . . . Bonder, M. J. (2017). An epigenome-wide association study meta-analysis of educational attainment. Mol. Psychiatr., 22(12), 1680-1690.

Küpers, L. K., Monnereau, C., Sharp, G. C., Yousefi, P., Salas, L. A., Ghantous, A., . . . Czamara, D. (2019). Meta-analysis of epigenome-wide association studies in neonates reveals widespread differential DNA methylation associated with birthweight. Nat. Commun., 10(1), 1893.

Lee, K. W. K., Richmond, R., Hu, P., French, L., Shin, J., Bourdon, C., . . . Gaunt, T. (2015). Prenatal exposure to maternal cigarette smoking and DNA methylation: epigenome-wide association in a discovery sample of adolescents and replication in an independent cohort at birth through 17 years of age. Environmental health perspective

Ligthart, S., Marzi, C., Aslibekyan, S., Mendelson, M. M., Conneely, K. N., Tanaka, T., . . . Guan, W. (2016). DNA methylation signatures of chronic low-grade inflammation are associated with complex diseases. Genome Biol., 17(1), 255.

Liu, C., Marioni, R. E., Hedman, Å. K., Pfeiffer, L., Tsai, P.-C., Reynolds, L. M., . . . Tanaka, T. (2018). A DNA methylation biomarker of alcohol consumption. Mol. Psychiatr., 23(2), 422-433.

Logue, M. W., Miller, M. W., Wolf, E. J., Huber, B. R., Morrison, F. G., Zhou, Z., . . . Ratanatharathorn, A. (2020). An epigenome-wide association study of posttraumatic stress disorder in US veterans implicates several new DNA methylation loci. Clinical epigenetics, 12, 1-14.

Marioni, R. E., McRae, A. F., Bressler, J., Colicino, E., Hannon, E., Li, S., . . . Tsai, P.-C. (2018). Meta-analysis of epigenome-wide association studies of cognitive abilities. Mol. Psychiatr., 23(11), 2133-2144.

Markunas, C. A., Xu, Z., Harlid, S., Wade, P. A., Lie, R. T., Taylor, J. A., & Wilcox, A. J. (2014). Identification of DNA methylation changes in newborns related to maternal smoking during pregnancy. Environmental health perspectives, 122(10), 1147-1153.

Marzi, S. J., Sugden, K., Arseneault, L., Belsky, D. W., Burrage, J., Corcoran, D. L., . . . Moffitt, T. E. (2018). Analysis of DNA methylation in young people: limited evidence for an association between victimization stress and epigenetic variation in blood. Am. J. Psychiat., 175(6), 517-529.

Monick, M. M., Beach, S. R. H., Plume, J., Sears, R., Gerrard, M., Brody, G. H., & Philibert, R. A. (2012). Coordinated changes in AHRR methylation in lymphoblasts and pulmonary macrophages from smokers. American Journal of Medical Genetics Part B: Neuropsychiatric Genetics, 159(2), 141-151.

Mulder, R. H., Neumann, A., Cecil, C. A. M., Walton, E., Houtepen, L. C., Simpkin, A. J., . . . Felix, J. F. (2021). Epigenome-wide change and variation in DNA methylation in childhood: Trajectories from birth to late adolescence. Human molecular genetics.

Ochoa-Rosales, C., Portilla-Fernandez, E., Nano, J., Wilson, R., Lehne, B., Mishra, P. P., . . . Juvinao-Quintero, D. (2020). Epigenetic link between statin therapy and type 2 diabetes. Diabetes Care, 43(4), 875-884.

Petersen, A.-K., Zeilinger, S., Kastenmüller, G., Römisch-Margl, W., Brugger, M., Peters, A., . . . Pagel, P. (2014). Epigenetics meets metabolomics: an epigenome-wide association study with blood serum metabolic traits. Human molecular genetics, 23(2), 534-545.

Philibert, R. A., Beach, S. R. H., & Brody, G. H. (2012). Demethylation of the aryl hydrocarbon receptor repressor as a biomarker for nascent smokers. Epigenetics, 7(11), 1331-1338.

Portales-Casamar, E., Lussier, A. A., Jones, M. J., MacIsaac, J. L., Edgar, R. D., Mah, S. M., . . . Cynader, M. S. (2016). DNA methylation signature of human fetal alcohol spectrum disorder. Epigenetics & chromatin, 9, 1-20.

Portilla-Fernández, E., Hwang, S.-J., Wilson, R., Maddock, J., Hill, W. D., Teumer, A., . . . Ligthart, S. (2021). Meta-analysis of epigenome-wide association studies of carotid intima-media thickness. European journal of epidemiology, 36, 1143-1155.

Richmond, R. C., Sillero-Rejon, C., Khouja, J. N., Prince, C., Board, A., Sharp, G., . . . Gage, S. H. (2021). Investigating the DNA methylation profile of e-cigarette use. Clinical epigenetics, 13, 1-13.

Richmond, R. C., Simpkin, A. J., Woodward, G., Gaunt, T. R., Lyttleton, O., McArdle, W. L., . . . Tilling, K. (2014). Prenatal exposure to maternal smoking and offspring DNA methylation across the lifecourse: findings from the Avon Longitudinal Study of Parents and Children (ALSPAC). Human molecular genetics, 24(8), 2201-2217.

Rzehak, P., Saffery, R., Reischl, E., Covic, M., Wahl, S., Grote, V., . . . Closa-Monasterolo, R. (2016). Maternal smoking during pregnancy and DNA-methylation in children at age 5.5 years: epigenome-wide-analysis in the European Childhood Obesity Project (CHOP)-Study. PLoS ONE, 11(5), e0155554.

Sayols-Baixeras, S., Lluís-Ganella, C., Subirana, I., Salas, L. A., Vilahur, N., Corella, D., . . . Moran, S. (2015). Identification of a new locus and validation of previously reported loci showing differential methylation associated with smoking. The REGICOR study. Epigenetics, 10(12), 1156-1165.

Shah, S., McRae, A. F., Marioni, R. E., Harris, S. E., Gibson, J., Henders, A. K., . . . Corley, J. (2014). Genetic and environmental exposures constrain epigenetic drift over the human life course. Genome research, 24(11), 1725-1733.

Shenker, N. S., Polidoro, S., van Veldhoven, K., Sacerdote, C., Ricceri, F., Birrell, M. A., . . . Flanagan, J. M. (2013). Epigenome-wide association study in the European Prospective Investigation into Cancer and Nutrition (EPIC-Turin) identifies novel genetic loci associated with smoking. Human molecular genetics, 22(5), 843-851.

Sikdar, S., Joehanes, R., Joubert, B. R., Xu, C.-J., Vives-Usano, M., Rezwan, F. I., . . . Richmond, R. C. (2019). Comparison of smoking-related DNA methylation between newborns from prenatal exposure and adults from personal smoking. Epigenomics, 11(13), 1487-1500.

Singmann, P., Shem-Tov, D., Wahl, S., Grallert, H., Fiorito, G., Shin, S.-Y., . . . Baran, Y. (2015). Characterization of whole-genome autosomal differences of DNA methylation between men and women. Epigenetics & chromatin, 8(1), 43.

Smith, A. K., Conneely, K. N., Kilaru, V., Mercer, K. B., Weiss, T. E., Bradley, B., . . . Ressler, K. J. (2011). Differential immune system DNA methylation and cytokine regulation in post-traumatic stress disorder. Am J Med Genet Part B Neuropsychiatr Genet, 156(6), 700-708. doi:10.1002/ajmg.b.31212

Stephenson, M., Bollepalli, S., Cazaly, E., Salvatore, J. E., Barr, P., Rose, R. J., . . . Ollikainen, M. (2021). Associations of alcohol consumption with epigenome-wide DNA methylation and epigenetic age acceleration: individual-level and co-twin comparison analyses. Alcoholism: Clinical and Experimental Research, 45(2), 318-328.

Sun, Y.-Q., Richmond, R. C., Suderman, M., Min, J. L., Battram, T., Flatberg, A., . . . Jiang, L. (2021). Assessing the role of genome-wide DNA methylation between smoking and risk of lung cancer using repeated measurements: the HUNT study. Int. J. Epidemiol., 50(5), 1482-1497.

Terzikhan, N., Xu, H., Edris, A., Bracke, K. R., Verhamme, F. M., Stricker, B. H. C., . . . Brusselle, G. G. (2021). Epigenome-wide association study on diffusing capacity of the lung. ERJ Open Research, 7(1).

Teschendorff, A. E., Yang, Z., Wong, A., Pipinikas, C. P., Jiao, Y., Jones, A., . . . Thirlwell, C. (2015). Correlation of smoking-associated DNA methylation changes in buccal cells with DNA methylation changes in epithelial cancer. JAMA oncology, 1(4), 476-485.

Tsaprouni, L. G., Yang, T.-P., Bell, J., Dick, K. J., Kanoni, S., Nisbet, J., . . . Meduri, E. (2014). Cigarette smoking reduces DNA methylation levels at multiple genomic loci but the effect is partially reversible upon cessation. Epigenetics, 9(10), 1382-1396.

van Dongen, J., Bonder, M. J., Dekkers, K. F., Nivard, M. G., van Iterson, M., Willemsen, G., . . . Franke, L. (2018). DNA methylation signatures of educational attainment. NPJ science of learning, 3(1), 7.

van Dongen, J., Hagenbeek, F. A., Suderman, M., Roetman, P. J., Sugden, K., Chiocchetti, A. G., . . . Adams, M. J. (2021). DNA methylation signatures of aggression and closely related constructs: A meta-analysis of epigenome-wide studies across the lifespan. Mol. Psychiatr., 26(6), 2148-2162.

Wahl, A., Kasela, S., Carnero-Montoro, E., van Iterson, M., Štambuk, J., Sharma, S., . . . Razdorov, G. (2018). IgG glycosylation and DNA methylation are interconnected with smoking. Biochimica et Biophysica Acta (BBA)-General Subjects, 1862(3), 637-648.

Wiklund, P., Karhunen, V., Richmond, R. C., Parmar, P., Rodriguez, A., De Silva, M., . . . Veijola, J. (2019). DNA methylation links prenatal smoking exposure to later life health outcomes in offspring. Clinical epigenetics, 11, 1-16.

Wozniak, M. B., Le Calvez-Kelm, F., Abedi-Ardekani, B., Byrnes, G., Durand, G., Carreira, C., . . . Foretova, L. (2013). Integrative genome-wide gene expression profiling of clear cell renal cell carcinoma in Czech Republic and in the United States. PLoS ONE, 8(3), e57886.

Xu, R., Hong, X., Zhang, B., Huang, W., Hou, W., Wang, G., . . . Ji, H. (2021). DNA methylation mediates the effect of maternal smoking on offspring birthweight: a birth cohort study of multi-ethnic US mother–newborn pairs. Clinical epigenetics, 13, 1-13.

Zaghlool, S. B., Al-Shafai, M., Al Muftah, W. A., Kumar, P., Falchi, M., & Suhre, K. (2015). Association of DNA methylation with age, gender, and smoking in an Arab population. Clinical epigenetics, 7, 1-12.

Zeilinger, S., Kühnel, B., Klopp, N., Baurecht, H., Kleinschmidt, A., Gieger, C., . . . Peters, A. (2013). Tobacco smoking leads to extensive genome-wide changes in DNA methylation. PLoS ONE, 8(5), e63812.

Zhang, Y., Florath, I., Saum, K.-U., & Brenner, H. (2016). Self-reported smoking, serum cotinine, and blood DNA methylation. Environ. Res., 146, 395-403.

Zhao, N., Ruan, M., Koestler, D. C., Lu, J., Marsit, C. J., Kelsey, K. T., . . . Michaud, D. S. (2022). Epigenome-wide scan identifies differentially methylated regions for lung cancer using pre-diagnostic peripheral blood. Epigenetics, 17(4), 460-472.

Zhu, X., Li, J., Deng, S., Yu, K., Liu, X., Deng, Q., . . . Guo, H. (2016). Genome-wide analysis of DNA methylation and cigarette smoking in a Chinese population. Environmental health perspectives, 124(7), 966-973.
