# Supplementary material for: Interactive effects of genotype with prenatal stress on DNA methylation at birth
Source: Mol Psychiatry. 2025 Oct 24;30(12):5749–59. doi: 10.1038/s41380-025-03312-6 (PMC12602324; doi:10.1038/s41380-025-03312-6)
Supplement: Supplementary file 5 — SM Table 2 [file 41380_2025_3312_MOESM5_ESM.pdf]

Supplemental Table 2. GxEmodel associations per stressor

SNP	CpG	Life events					Contextual stress					Personal stress					Interpersonal stress				
		B	SE	*p*	Direction	I^2	B	SE	*p*	Direction	I^2	B	SE	*p*	Direction	I^2	B	SE	*p*	Direction	I^2
rs12901653	cg24317086	-0.0002	0.0011	0.8629	++-	0	-0.0021	0.0012	0.07429	---	0	0.0019	0.0012	0.1085	+++	30.2	0.001	0.0012	0.3904	++-	0
rs76542426	cg06592260	-0.0005	0.0006	0.4577	+--	0	0.0003	0.0006	0.6859	++-	0	-0.0004	0.0006	0.5651	---	0	0.0007	0.0006	0.2599	-++	46.2
rs10279675	cg06592260	-0.0004	0.0006	0.4665	+--	0	0.0003	0.0006	0.6791	++-	0	-0.0004	0.0006	0.5581	---	0	0.0007	0.0006	0.2526	-++	47.3
rs2188287	cg06592260	-0.0004	0.0006	0.4799	+--	0	0.0003	0.0006	0.6821	++-	0	-0.0004	0.0006	0.5478	---	0	0.0007	0.0006	0.2716	-++	48.2
rs10251976	cg06592260	-0.0004	0.0006	0.463	+--	0	0.0002	0.0006	0.712	++-	0	-0.0004	0.0006	0.5263	---	0	0.0007	0.0006	0.2773	-++	51.8

Estimates for the different stressors are mutually adjusted for each other, i.e. a single regression was performed for each SNP-CpG combination

Direction indicates direction of estimate for *GENR 450K* , *GENR EPIC* , and *ALSPAC 450K* , respectively
