# Supplementary material for: Interactive effects of genotype with prenatal stress on DNA methylation at birth
Source: Mol Psychiatry. 2025 Oct 24;30(12):5749–59. doi: 10.1038/s41380-025-03312-6 (PMC12602324; doi:10.1038/s41380-025-03312-6)
Supplement: Supplementary file 6 — SM Table 3 [file 41380_2025_3312_MOESM6_ESM.pdf]

Supplemental Table 3. Emodel associations per stressor

CpG	**Life events**					**Contextual stress**					**Personal stress**					**Interpersonal stress**				
	B	SE	*p*	Direction	I^2	B	SE	*p*	Direction	I^2	B	SE	*p*	Direction	I^2	B	SE	*p*	Direction	I^2
cg05575921	-0.0007	0.001	0.4602	--+	0	-0.0078	0.001	**4.42E-14**	---	79.2	-0.001	0.001	3.39E-01	-++	69.6	-0.0028	0.001	**7.53E-03**	---	54.3
cg09935388	-0.0026	0.0024	0.2876	-+-	0	-0.0145	0.0025	**6.18E-09**	---	0	0.0003	0.0025	8.88E-01	--+	67.4	-0.007	0.0025	**5.44E-03**	---	32.1
cg04180046	0.0015	0.0013	0.2283	++-	5.7	0.0053	0.0013	**7.36E-05**	+++	0	0.0026	0.0013	5.02E-02	+++	0	0.0008	0.0013	0.5776	-++	6.8

Estimates for the different stressors are mutually adjusted for each other, i.e. a single regression was performed for each CpG

Direction indicates direction of estimate for *GENR 450K* , *GENR EPIC* , and *ALSPAC 450K* , respectively
