# Supplementary material for: Interactive effects of genotype with prenatal stress on DNA methylation at birth
Source: Mol Psychiatry. 2025 Oct 24;30(12):5749–59. doi: 10.1038/s41380-025-03312-6 (PMC12602324; doi:10.1038/s41380-025-03312-6)
Supplement: Supplementary file 7 — SM Table 4 [file 41380_2025_3312_MOESM7_ESM.pdf]

**Supplemental Table 4.** Gene Ontology enrichment results for genome-wide SNPs in GxEmodel

| Ontology | GO ID | Term | *P* -value overrepresentation | FWER overrepresentation | *P* -value underrepresentation | FWER underrepresentation |
|---|---|---|---|---|---|---|
| cellular component | GO:0030054 | cell junction | 1.88E-16 | 0.000 | 1.00E+00 | 1.000 |
| biological process | GO:0007399 | nervous system development | 1.45E-15 | 0.000 | 1.00E+00 | 1.000 |
| cellular component | GO:0071944 | cell periphery | 1.51E-15 | 0.000 | 1.00E+00 | 1.000 |
| biological process | GO:0031175 | neuron projection development | 2.58E-14 | 0.000 | 1.00E+00 | 1.000 |
| biological process | GO:0048666 | neuron development | 5.19E-14 | 0.000 | 1.00E+00 | 1.000 |
| cellular component | GO:0005886 | plasma membrane | 9.18E-14 | 0.000 | 1.00E+00 | 1.000 |
| biological process | GO:0048731 | system development | 1.13E-13 | 0.000 | 1.00E+00 | 1.000 |
| biological process | GO:0048699 | generation of neurons | 1.18E-13 | 0.000 | 1.00E+00 | 1.000 |
| biological process | GO:0034330 | cell junction organization | 3.44E-13 | 0.000 | 1.00E+00 | 1.000 |
| biological process | GO:0022008 | neurogenesis | 4.84E-13 | 0.000 | 1.00E+00 | 1.000 |
| biological process | GO:0032990 | cell part morphogenesis | 5.81E-13 | 0.000 | 1.00E+00 | 1.000 |
| cellular component | GO:0045202 | synapse | 6.30E-13 | 0.000 | 1.00E+00 | 1.000 |
| biological process | GO:0009653 | anatomical structure morphogenesis | 1.21E-12 | 0.000 | 1.00E+00 | 1.000 |
| biological process | GO:0030182 | neuron differentiation | 1.26E-12 | 0.000 | 1.00E+00 | 1.000 |
| biological process | GO:0007275 | multicellular organism development | 1.52E-12 | 0.000 | 1.00E+00 | 1.000 |
| biological process | GO:0048468 | cell development | 1.82E-12 | 0.000 | 1.00E+00 | 1.000 |
| biological process | GO:0030030 | cell projection organization | 2.38E-12 | 0.000 | 1.00E+00 | 1.000 |
| biological process | GO:0048856 | anatomical structure development | 2.96E-12 | 0.000 | 1.00E+00 | 1.000 |
| biological process | GO:0048858 | cell projection morphogenesis | 4.00E-12 | 0.000 | 1.00E+00 | 1.000 |
| biological process | GO:0120039 | plasma membrane bounded cell projection morphogenesis | 4.80E-12 | 0.000 | 1.00E+00 | 1.000 |
| biological process | GO:0120036 | plasma membrane bounded cell projection organization | 5.18E-12 | 0.000 | 1.00E+00 | 1.000 |
| biological process | GO:0032502 | developmental process | 7.05E-12 | 0.000 | 1.00E+00 | 1.000 |
| biological process | GO:0050808 | synapse organization | 9.31E-12 | 0.000 | 1.00E+00 | 1.000 |
| biological process | GO:0048812 | neuron projection morphogenesis | 9.40E-12 | 0.000 | 1.00E+00 | 1.000 |
| biological process | GO:0048869 | cellular developmental process | 1.49E-11 | 0.000 | 1.00E+00 | 1.000 |
| biological process | GO:0030154 | cell differentiation | 1.50E-11 | 0.000 | 1.00E+00 | 1.000 |
| cellular component | GO:0097060 | synaptic membrane | 2.81E-11 | 0.000 | 1.00E+00 | 1.000 |
| biological process | GO:0006928 | movement of cell or subcellular component | 3.64E-11 | 0.000 | 1.00E+00 | 1.000 |
| biological process | GO:0032989 | cellular component morphogenesis | 3.84E-11 | 0.000 | 1.00E+00 | 1.000 |
| biological process | GO:0034329 | cell junction assembly | 4.38E-11 | 0.000 | 1.00E+00 | 1.000 |
| cellular component | GO:0043005 | neuron projection | 5.23E-11 | 0.000 | 1.00E+00 | 1.000 |
| biological process | GO:0032501 | multicellular organismal process | 6.43E-11 | 0.000 | 1.00E+00 | 1.000 |
| cellular component | GO:0098794 | postsynapse | 1.10E-10 | 0.000 | 1.00E+00 | 1.000 |
| biological process | GO:0007411 | axon guidance | 1.34E-10 | 0.000 | 1.00E+00 | 1.000 |
| biological process | GO:0097485 | neuron projection guidance | 1.34E-10 | 0.000 | 1.00E+00 | 1.000 |
| cellular component | GO:0032279 | asymmetric synapse | 1.37E-10 | 0.000 | 1.00E+00 | 1.000 |
| biological process | GO:0007267 | cell-cell signaling | 1.61E-10 | 0.000 | 1.00E+00 | 1.000 |
| biological process | GO:0000902 | cell morphogenesis | 2.35E-10 | 0.000 | 1.00E+00 | 1.000 |
| biological process | GO:0007416 | synapse assembly | 5.68E-10 | 0.000 | 1.00E+00 | 1.000 |
| cellular component | GO:0099572 | postsynaptic specialization | 5.71E-10 | 0.000 | 1.00E+00 | 1.000 |
| cellular component | GO:0016020 | membrane | 6.23E-10 | 0.000 | 1.00E+00 | 1.000 |
| biological process | GO:0031344 | regulation of cell projection organization | 7.77E-10 | 0.000 | 1.00E+00 | 1.000 |
| cellular component | GO:0014069 | postsynaptic density | 7.94E-10 | 0.000 | 1.00E+00 | 1.000 |
| biological process | GO:0050804 | modulation of chemical synaptic transmission | 8.46E-10 | 0.000 | 1.00E+00 | 1.000 |
| cellular component | GO:0098984 | neuron to neuron synapse | 8.71E-10 | 0.000 | 1.00E+00 | 1.000 |
| biological process | GO:0099177 | regulation of trans-synaptic signaling | 9.62E-10 | 0.000 | 1.00E+00 | 1.000 |
| cellular component | GO:0042995 | cell projection | 1.70E-09 | 0.000 | 1.00E+00 | 1.000 |
| cellular component | GO:0097447 | dendritic tree | 3.64E-09 | 0.000 | 1.00E+00 | 1.000 |
| biological process | GO:0120035 | regulation of plasma membrane bounded cell projection organization | 4.70E-09 | 0.000 | 1.00E+00 | 1.000 |
| biological process | GO:0099537 | trans-synaptic signaling | 4.94E-09 | 0.000 | 1.00E+00 | 1.000 |
| biological process | GO:0007268 | chemical synaptic transmission | 5.39E-09 | 0.000 | 1.00E+00 | 1.000 |
| biological process | GO:0098916 | anterograde trans-synaptic signaling | 5.39E-09 | 0.000 | 1.00E+00 | 1.000 |
| biological process | GO:0099536 | synaptic signaling | 5.53E-09 | 0.000 | 1.00E+00 | 1.000 |

| | | | | | | |
|---|---|---|---|---|---|---|
| cellular component | GO:0030425 | dendrite | 6.01E-09 | 0.000 | 1.00E+00 | 1.000 |
| cellular component | GO:0098590 | plasma membrane region | 1.46E-08 | 0.000 | 1.00E+00 | 1.000 |
| cellular component | GO:0005911 | cell-cell junction | 1.54E-08 | 0.000 | 1.00E+00 | 1.000 |
| cellular component | GO:0120025 | plasma membrane bounded cell projection | 1.67E-08 | 0.000 | 1.00E+00 | 1.000 |
| cellular component | GO:0045211 | postsynaptic membrane | 1.89E-08 | 0.000 | 1.00E+00 | 1.000 |
| cellular component | GO:0005912 | adherens junction | 1.20E-07 | 0.000 | 1.00E+00 | 1.000 |
| cellular component | GO:0098978 | glutamatergic synapse | 1.55E-07 | 0.000 | 1.00E+00 | 1.000 |
| cellular component | GO:0099634 | postsynaptic specialization membrane | 1.82E-07 | 0.000 | 1.00E+00 | 1.000 |
| cellular component | GO:0098793 | presynapse | 3.01E-07 | 0.000 | 1.00E+00 | 1.000 |
| cellular component | GO:0036477 | somatodendritic compartment | 3.31E-07 | 0.000 | 1.00E+00 | 1.000 |
| cellular component | GO:0098839 | postsynaptic density membrane | 3.70E-07 | 0.000 | 1.00E+00 | 1.000 |
| cellular component | GO:0070161 | anchoring junction | 4.08E-07 | 0.000 | 1.00E+00 | 1.000 |
| cellular component | GO:0034703 | cation channel complex | 4.44E-07 | 0.000 | 1.00E+00 | 1.000 |
| cellular component | GO:0034702 | ion channel complex | 6.88E-07 | 0.000 | 1.00E+00 | 1.000 |
| biological process | GO:0050807 | regulation of synapse organization | 8.29E-09 | 0.001 | 1.00E+00 | 1.000 |
| biological process | GO:0008038 | neuron recognition | 1.07E-08 | 0.001 | 1.00E+00 | 1.000 |
| biological process | GO:0040011 | locomotion | 1.69E-08 | 0.001 | 1.00E+00 | 1.000 |
| biological process | GO:0098742 | cell-cell adhesion via plasma-membrane adhesion molecules | 1.89E-08 | 0.001 | 1.00E+00 | 1.000 |
| biological process | GO:0050803 | regulation of synapse structure or activity | 2.45E-08 | 0.001 | 1.00E+00 | 1.000 |
| cellular component | GO:1990351 | transporter complex | 3.11E-06 | 0.001 | 1.00E+00 | 1.000 |
| biological process | GO:0010646 | regulation of cell communication | 3.44E-08 | 0.002 | 1.00E+00 | 1.000 |
| biological process | GO:0010975 | regulation of neuron projection development | 3.92E-08 | 0.002 | 1.00E+00 | 1.000 |
| biological process | GO:0016477 | cell migration | 4.24E-08 | 0.002 | 1.00E+00 | 1.000 |
| biological process | GO:0048588 | developmental cell growth | 4.62E-08 | 0.002 | 1.00E+00 | 1.000 |
| biological process | GO:0023051 | regulation of signaling | 5.28E-08 | 0.002 | 1.00E+00 | 1.000 |
| biological process | GO:0060560 | developmental growth involved in morphogenesis | 6.71E-08 | 0.002 | 1.00E+00 | 1.000 |
| biological process | GO:0061564 | axon development | 6.73E-08 | 0.002 | 1.00E+00 | 1.000 |
| biological process | GO:0007409 | axonogenesis | 7.09E-08 | 0.002 | 1.00E+00 | 1.000 |
| biological process | GO:0032879 | regulation of localization | 7.48E-08 | 0.002 | 1.00E+00 | 1.000 |
| biological process | GO:0051963 | regulation of synapse assembly | 7.55E-08 | 0.002 | 1.00E+00 | 1.000 |
| biological process | GO:0065008 | regulation of biological quality | 8.96E-08 | 0.002 | 1.00E+00 | 1.000 |
| biological process | GO:0022610 | biological adhesion | 1.20E-07 | 0.002 | 1.00E+00 | 1.000 |
| biological process | GO:0007155 | cell adhesion | 1.32E-07 | 0.003 | 1.00E+00 | 1.000 |
| biological process | GO:0048667 | cell morphogenesis involved in neuron differentiation | 1.34E-07 | 0.003 | 1.00E+00 | 1.000 |
| biological process | GO:0006935 | chemotaxis | 1.43E-07 | 0.003 | 1.00E+00 | 1.000 |
| biological process | GO:0051179 | localization | 1.83E-07 | 0.003 | 1.00E+00 | 1.000 |
| biological process | GO:0003008 | system process | 1.87E-07 | 0.003 | 1.00E+00 | 1.000 |
| biological process | GO:0031346 | positive regulation of cell projection organization | 1.95E-07 | 0.003 | 1.00E+00 | 1.000 |
| biological process | GO:0048589 | developmental growth | 2.17E-07 | 0.003 | 1.00E+00 | 1.000 |
| biological process | GO:0042330 | taxis | 2.30E-07 | 0.003 | 1.00E+00 | 1.000 |
| biological process | GO:0035249 | synaptic transmission, glutamatergic | 2.68E-07 | 0.003 | 1.00E+00 | 1.000 |
| biological process | GO:0051270 | regulation of cellular component movement | 2.90E-07 | 0.003 | 1.00E+00 | 1.000 |
| biological process | GO:0098609 | cell-cell adhesion | 3.52E-07 | 0.003 | 1.00E+00 | 1.000 |
| biological process | GO:0030334 | regulation of cell migration | 4.51E-07 | 0.003 | 1.00E+00 | 1.000 |
| biological process | GO:0099054 | presynapse assembly | 4.69E-07 | 0.003 | 1.00E+00 | 1.000 |
| biological process | GO:0040012 | regulation of locomotion | 6.84E-07 | 0.003 | 1.00E+00 | 1.000 |
| biological process | GO:0007154 | cell communication | 7.59E-07 | 0.005 | 1.00E+00 | 1.000 |
| biological process | GO:0009887 | animal organ morphogenesis | 7.60E-07 | 0.005 | 1.00E+00 | 1.000 |
| cellular component | GO:1902495 | transmembrane transporter complex | 1.46E-05 | 0.005 | 1.00E+00 | 1.000 |
| cellular component | GO:0005891 | voltage-gated calcium channel complex | 1.64E-05 | 0.005 | 1.00E+00 | 1.000 |
| cellular component | GO:0030424 | axon | 1.71E-05 | 0.005 | 1.00E+00 | 1.000 |
| molecular function | GO:0008066 | glutamate receptor activity | 6.71E-06 | 0.006 | 1.00E+00 | 1.000 |
| biological process | GO:2000145 | regulation of cell motility | 1.06E-06 | 0.007 | 1.00E+00 | 1.000 |
| biological process | GO:0000904 | cell morphogenesis involved in differentiation | 1.08E-06 | 0.008 | 1.00E+00 | 1.000 |
| biological process | GO:0023052 | signaling | 1.10E-06 | 0.008 | 1.00E+00 | 1.000 |

| | | | | | | |
|---|---|---|---|---|---|---|
| biological process | GO:0099172 | presynapse organization | 1.15E-06 | 0.008 | 1.00E+00 | 1.000 |
| biological process | GO:1990138 | neuron projection extension | 1.51E-06 | 0.008 | 1.00E+00 | 1.000 |
| biological process | GO:0050793 | regulation of developmental process | 1.65E-06 | 0.008 | 1.00E+00 | 1.000 |
| molecular function | GO:0015267 | channel activity | 1.26E-05 | 0.008 | 1.00E+00 | 1.000 |
| biological process | GO:0001764 | neuron migration | 1.79E-06 | 0.009 | 1.00E+00 | 1.000 |
| cellular component | GO:0042734 | presynaptic membrane | 1.97E-05 | 0.009 | 1.00E+00 | 1.000 |
| molecular function | GO:0022803 | passive transmembrane transporter activity | 1.38E-05 | 0.010 | 1.00E+00 | 1.000 |
| molecular function | GO:0005216 | ion channel activity | 1.39E-05 | 0.010 | 1.00E+00 | 1.000 |
| biological process | GO:1901888 | regulation of cell junction assembly | 3.06E-06 | 0.014 | 1.00E+00 | 1.000 |
| biological process | GO:0051049 | regulation of transport | 3.06E-06 | 0.014 | 1.00E+00 | 1.000 |
| biological process | GO:0048513 | animal organ development | 3.15E-06 | 0.014 | 1.00E+00 | 1.000 |
| biological process | GO:0051966 | regulation of synaptic transmission, glutamatergic | 3.38E-06 | 0.014 | 1.00E+00 | 1.000 |
| biological process | GO:0048870 | cell motility | 3.48E-06 | 0.014 | 1.00E+00 | 1.000 |
| biological process | GO:0051674 | localization of cell | 3.48E-06 | 0.014 | 1.00E+00 | 1.000 |
| biological process | GO:0040007 | growth | 3.72E-06 | 0.014 | 1.00E+00 | 1.000 |
| molecular function | GO:0022843 | voltage-gated cation channel activity | 2.31E-05 | 0.014 | 1.00E+00 | 1.000 |
| molecular function | GO:0022836 | gated channel activity | 2.43E-05 | 0.014 | 1.00E+00 | 1.000 |
| molecular function | GO:0008013 | beta-catenin binding | 2.51E-05 | 0.014 | 1.00E+00 | 1.000 |
| molecular function | GO:0005262 | calcium channel activity | 2.74E-05 | 0.014 | 1.00E+00 | 1.000 |
| biological process | GO:0007417 | central nervous system development | 4.68E-06 | 0.017 | 1.00E+00 | 1.000 |
| biological process | GO:0007156 | homophilic cell adhesion via plasma membrane adhesion molecules | 5.13E-06 | 0.017 | 1.00E+00 | 1.000 |
| molecular function | GO:0005261 | cation channel activity | 3.35E-05 | 0.017 | 1.00E+00 | 1.000 |
| biological process | GO:0050954 | sensory perception of mechanical stimulus | 5.77E-06 | 0.018 | 1.00E+00 | 1.000 |
| biological process | GO:0022603 | regulation of anatomical structure morphogenesis | 6.14E-06 | 0.019 | 1.00E+00 | 1.000 |
| cellular component | GO:0034704 | calcium channel complex | 8.44E-05 | 0.026 | 1.00E+00 | 1.000 |
| molecular function | GO:0004970 | ionotropic glutamate receptor activity | 4.47E-05 | 0.028 | 1.00E+00 | 1.000 |
| biological process | GO:0007158 | neuron cell-cell adhesion | 7.99E-06 | 0.029 | 1.00E+00 | 1.000 |
| biological process | GO:0051094 | positive regulation of developmental process | 8.21E-06 | 0.030 | 1.00E+00 | 1.000 |
| biological process | GO:0051239 | regulation of multicellular organismal process | 8.62E-06 | 0.030 | 1.00E+00 | 1.000 |
| molecular function | GO:0098631 | cell adhesion mediator activity | 6.93E-05 | 0.037 | 1.00E+00 | 1.000 |
| biological process | GO:1901890 | positive regulation of cell junction assembly | 1.30E-05 | 0.039 | 1.00E+00 | 1.000 |
| molecular function | GO:0008331 | high voltage-gated calcium channel activity | 7.54E-05 | 0.039 | 1.00E+00 | 1.000 |
| molecular function | GO:0005244 | voltage-gated ion channel activity | 8.63E-05 | 0.041 | 1.00E+00 | 1.000 |
| molecular function | GO:0022832 | voltage-gated channel activity | 8.63E-05 | 0.041 | 1.00E+00 | 1.000 |
| cellular component | GO:0044297 | cell body | 1.01E-04 | 0.042 | 1.00E+00 | 1.000 |
| biological process | GO:0051962 | positive regulation of nervous system development | 1.57E-05 | 0.046 | 1.00E+00 | 1.000 |
| biological process | GO:0051240 | positive regulation of multicellular organismal process | 1.61E-05 | 0.046 | 1.00E+00 | 1.000 |
| molecular function | GO:0003676 | nucleic acid binding | 1.00E+00 | 1.000 | 4.83E-04 | 0.047 |
| cellular component | GO:0005622 | intracellular anatomical structure | 1.00E+00 | 1.000 | 3.94E-04 | 0.048 |
| cellular component | GO:0005739 | mitochondrion | 1.00E+00 | 1.000 | 3.40E-04 | 0.039 |
| cellular component | GO:0043231 | intracellular membrane-bounded organelle | 1.00E+00 | 1.000 | 1.56E-04 | 0.018 |
| molecular function | GO:0003723 | RNA binding | 1.00E+00 | 1.000 | 7.61E-05 | 0.001 |
| cellular component | GO:0005840 | ribosome | 1.00E+00 | 1.000 | 1.64E-04 | 0.018 |
| cellular component | GO:0031974 | membrane-enclosed lumen | 1.00E+00 | 1.000 | 3.37E-05 | 0.008 |
| cellular component | GO:0043233 | organelle lumen | 1.00E+00 | 1.000 | 3.37E-05 | 0.008 |
| cellular component | GO:0070013 | intracellular organelle lumen | 1.00E+00 | 1.000 | 3.37E-05 | 0.008 |
| biological process | GO:0006974 | cellular response to DNA damage stimulus | 1.00E+00 | 1.000 | 2.62E-05 | 0.023 |
| biological process | GO:0006259 | DNA metabolic process | 1.00E+00 | 1.000 | 1.60E-05 | 0.013 |
| biological process | GO:0006281 | DNA repair | 1.00E+00 | 1.000 | 4.19E-06 | 0.000 |

FWER: family-wise error rate
