# Supplementary material for: Interactive effects of genotype with prenatal stress on DNA methylation at birth
Source: Mol Psychiatry. 2025 Oct 24;30(12):5749–59. doi: 10.1038/s41380-025-03312-6 (PMC12602324; doi:10.1038/s41380-025-03312-6)
Supplement: Supplementary file 8 — SM Table 5 [file 41380_2025_3312_MOESM8_ESM.pdf]

Supplemental Table 5. Gene Ontology enrichment results for genome-wide CpGs in GxEmodel

Ontology	GO ID	Term	*P* -value overrepresentation	FWER overrepresentation	*P* -value underrepresentation	FWER underrepresentation
biological process	GO:0007399	nervous system development	3.37E-10	0.000	1.00E+00	1.000
biological process	GO:0009653	anatomical structure morphogenesis	7.67E-09	0.000	1.00E+00	1.000
biological process	GO:0021675	nerve development	6.41E-08	0.000	1.00E+00	1.000
biological process	GO:0022008	neurogenesis	6.50E-08	0.000	1.00E+00	1.000
biological process	GO:0048699	generation of neurons	7.27E-08	0.000	1.00E+00	1.000
biological process	GO:0007417	central nervous system development	9.54E-08	0.000	1.00E+00	1.000
biological process	GO:0030182	neuron differentiation	1.49E-07	0.000	1.00E+00	1.000
biological process	GO:0048731	system development	1.50E-07	0.000	1.00E+00	1.000
biological process	GO:0007275	multicellular organism development	5.36E-07	0.000	1.00E+00	1.000
biological process	GO:0048598	embryonic morphogenesis	7.63E-07	0.000	1.00E+00	1.000
biological process	GO:0048856	anatomical structure development	8.28E-07	0.000	1.00E+00	1.000
biological process	GO:0072087	renal vesicle development	8.80E-07	0.000	1.00E+00	1.000
biological process	GO:0022603	regulation of anatomical structure morphogenesis	9.08E-07	0.000	1.00E+00	1.000
biological process	GO:0009887	animal organ morphogenesis	1.55E-06	0.002	1.00E+00	1.000
cellular component	GO:0043025	neuronal cell body	2.26E-05	0.004	1.00E+00	1.000
biological process	GO:0007423	sensory organ development	2.89E-06	0.005	1.00E+00	1.000
biological process	GO:0048666	neuron development	3.04E-06	0.005	1.00E+00	1.000
biological process	GO:0072210	metanephric nephron development	3.44E-06	0.006	1.00E+00	1.000
cellular component	GO:0043005	neuron projection	3.61E-05	0.006	1.00E+00	1.000
biological process	GO:0072273	metanephric nephron morphogenesis	3.46E-06	0.007	1.00E+00	1.000
biological process	GO:0072077	renal vesicle morphogenesis	4.09E-06	0.010	1.00E+00	1.000
biological process	GO:0048562	embryonic organ morphogenesis	4.18E-06	0.010	1.00E+00	1.000
biological process	GO:0030900	forebrain development	5.42E-06	0.010	1.00E+00	1.000
biological process	GO:0014033	neural crest cell differentiation	6.47E-06	0.010	1.00E+00	1.000
biological process	GO:0042474	middle ear morphogenesis	8.50E-06	0.020	1.00E+00	1.000
biological process	GO:0050793	regulation of developmental process	1.00E-05	0.021	1.00E+00	1.000
biological process	GO:0000902	cell morphogenesis	1.12E-05	0.021	1.00E+00	1.000
biological process	GO:0048706	embryonic skeletal system development	1.17E-05	0.022	1.00E+00	1.000
biological process	GO:0048568	embryonic organ development	1.34E-05	0.024	1.00E+00	1.000
biological process	GO:0048812	neuron projection morphogenesis	1.67E-05	0.033	1.00E+00	1.000
biological process	GO:0071625	vocalization behavior	1.81E-05	0.039	1.00E+00	1.000
biological process	GO:0048468	cell development	1.95E-05	0.043	1.00E+00	1.000
biological process	GO:1904888	cranial skeletal system development	2.12E-05	0.045	1.00E+00	1.000
biological process	GO:0040011	locomotion	2.16E-05	0.045	1.00E+00	1.000
biological process	GO:0021537	telencephalon development	2.24E-05	0.046	1.00E+00	1.000

FWER: family-wise error rate
